# Temporally resolved single-cell RNA sequencing reveals protective and pathological responses during herpes simplex virus CNS infection

**DOI:** 10.1186/s12974-025-03471-x

**Published:** 2025-05-31

**Authors:** Xiangning Ding, Xin Lai, Ida H. Klaestrup, Sara R. N. Jensen, Morten M. Nielsen, Kasper Thorsen, Marina Romero-Ramos, Yonglun Luo, Lin Lin, Line S. Reinert, Søren R. Paludan

**Affiliations:** 1 Department of Biomedicine, Aarhus University, Aarhus, Denmark; 2 Center for Immunology of Viral Infections, Aarhus University, Aarhus, Denmark; 3 DANDRITE, Aarhus University, Aarhus, Denmark; 4 Department of Molecular Medicine, Aarhus University Hospital, Aarhus, Denmark; 5 Steno Diabetes Center Aarhus, Aarhus University Hospital, Aarhus, Denmark

**Keywords:** Viral brain infection, Infection-induced brain inflammation, Single-cell RNA sequencing

## Abstract

**Background:**

Herpes Simplex Virus 1 (HSV-1) is a neurotropic virus causing encephalitis and post-infectious complications. Infections can induce a range of acute, subacute, and progressing brain disease, and in recent years it has emerged that immune responses are involved in the pathogenesis of these diseases.

**Methods:**

Mice were infected with HSV-1 through corneal infection, and the brain stem was analyzed using single-cell and GeoMx spatial transcriptomics. Through these technologies we profiled temporal transcriptomic changes in cell populations, pathways, and cell-cell communication associated with antiviral activity and inflammation-induced disturbance of physiological brain structures and activities.

**Results:**

We found that microglia proportions increased early after HSV-1 infection, followed by monocyte influx and later by T cells. The blood-brain barrier was disrupted, and transcriptomic profiles associated with homeostatic brain transcriptional activities were altered. Early transcriptional responses were dominated by antiviral and inflammatory activities. A microglia subpopulation with high type I interferon and chemokine expression localized to infection sites, likely mediating antiviral defense and immune recruitment. Monocyte subpopulations displayed a broader activation profile than microglia and was a central mediator of crosstalk between immune cells. Cytokines from microglia, monocytes, and T cells reprogrammed brain cells, notably endothelial cells and oligodendrocytes, disrupting brain functions. Comparing datasets from various brain diseases revealed the identified microglia subpopulation as specific to viral infections.

**Conclusions:**

This study identifies a unique population of virus-activated microglia with antiviral and proinflammatory properties and reveals monocytes to be a key driver of interactions driving pathology in the virus-infected brain.

**Supplementary Information:**

The online version contains supplementary material available at 10.1186/s12974-025-03471-x.

## Background

Viral infections of the central nervous system (CNS) are characterized by inflammation of the brain tissue, leading to a spectrum of symptoms ranging from mild headaches and fever to severe neurological impairments, such as seizures, memory loss, and potentially life-threatening complications [1]. Herpes Simplex Virus Type 1 (HSV-1) is highly prevalent in humans and the cause of herpes simplex encephalitis (HSE) [1, 2]. HSV-1 is a neurotropic virus that infects neurons in the sensory ganglia, spread to the CNS and potentially causing encephalitis [3]. However, if the virus is not limited by the peripheral immune system, HSV-1 can spread to the CNS, potentially causing encephalitis. Moreover, viral brain infections can prime for a variety of subacute brain conditions, and there are also epidemiological data linking viral infections to neurodegenerative diseases, as illustrated by the correlation between HSV-1 infection and development of Alzheimer’s disease [4]. Despite advancements in antiviral therapies, HSE and other infection-associated brain conditions continue to pose significant morbidity and mortality, highlighting the critical need for a deeper understanding of the underlying pathophysiological mechanisms.

In recent years, it has emerged that immune responses are involved in the pathogenesis of many brain diseases as well as in aging. This includes not only autoimmune diseases or acute infections but also numerous neurodegenerative diseases [5, 6]. Moreover, a range of brain conditions that can develop following infection are now known to have a strong immunological component [6, 7]. While the underlying mechanisms for this are complex and likely disease-specific, it is known that many key brain structures and cell types are highly sensitive to inflammatory activities. This includes the blood-brain barrier (BBB), neurons, and the myelination process [8–10]. Moreover, some cell types, including astrocytes and microglia, change phenotype in an inflammatory environment ultimately promoting neurodegeneration rather than providing support for the physiological processes of neurons [11]. Thus, understanding the nature and dynamics of immune responses in the brain and the interaction with physiological brain functions may provide information on central disease processes.

Viral brain infections are characterized by strong expression of the antiviral type I interferon (IFN, α/β) cytokines and a panel of inflammatory cytokines, such as Tumor necrosis factor (TNF) family members and interleukin (IL)1β and IL6 [12–14]. This is accompanied by influx of immune cells from the periphery with early recruitment of monocytes/macrophages and natural killer (NK) cells, and later T cells [15, 16]. Both brain resident and infiltrating cell types have been described to contribute to the innate immune response to viral CNS infections. For instance, microglia represent an important early source of IFNβ in the HSV-1-infected mouse brain [12, 17, 18], while in the case of a rhabdovirus infection, this was shown to be mainly derived from astrocytes [19]. Studies from human genetics and mouse models have shown that defects in the type I IFN system leads to susceptibility to HSE [20–24], thus highlighting the importance of this pathway in early host defense in the brain. The use of cell-type specific deficiency in IFN signaling has shown that the action of type I IFNs on neurons and astrocytes is essential to prevent disease development [25, 26]. Regarding the role of recruited immune cells in the virus-infected brain, different T cell subsets are known to play an important part in host defense [27, 28], largely due to type II IFN (IFN-γ)-driven antiviral activity [29]. Studies on how recruited monocytes/macrophages contribute to the control of brain infection with viruses, including HSV-1, have given conflicting results, suggesting both beneficial and deleterious effect on disease outcome [30–33].

As the brain is highly sensitive to immune-mediated activities, improved understanding of the immune response and its potential pathological activity in the context of HSE may provide new treatment options. Studies from various brain infection models have shown that inflammatory activity in the brain impairs brain development and function. For instance, upon West Nile virus (WNV) encephalitis infection in mice, T cells exert an IFN-γ-dependent response, which leads to microglial activation and cognitive dysfunction after eliminating the virus from the brain [7]. In the same model, impaired recruitment of Ly6c + monocytes into the brain prolonged survival [32]. In the context of HSV-1, Toll-like receptor 2-deficient mice are less susceptible to acute brain disease, correlating with lower expression of the inflammatory cytokine TNFα [34]. Data from a human brain organoid model have also shown the TNFα pathway to be highly upregulated upon HSV-1 infection and revealed that treatment with anti-inflammatory compounds prevented damage of neuronal processes and brain-associated epithelium [35]. The latter could indicate immune-mediated disruption of the BBB, which has been observed during infections with WNV and Japanese encephalitis virus (JEV) [16, 36]. Likewise, the detection of a range of markers in the cerebrospinal fluid from HSE patients could indicate BBB breakdown [37]. Thus, the inflammatory response in the virus-infected brain likely impacts both brain structure and function, but the mechanisms and dynamics are not well understood.

Single-cell technologies have emerged as powerful tools to perform deep phenotyping of cellular responses and interactions in complex biological processes in vivo. In the context of viral infections, this was first observed in the characterization of COVID-19 pathogenesis and enabled the identification of how recruited immune cells, notably monocytes, become hyper-inflammatory if recruited into a microenvironment with retained viral presence [38]. With respect to acute viral CNS infections, a panel of studies is starting to address the complexities of these diseases with omics technologies [16, 39, 40]. In one study on HSV-1 infection, CD11b + immune cells were isolated on day 6 post infection from the ventral posterolateral nucleus thalamic regions and sequenced. This led to the identification of a microglia subpopulation enriched for interleukin (IL)-1β signaling, apoptosis, and antigen presentation pathways [39]. However, there is a lack of detailed information on the brain immune response to HSV-1 infection at the single-cell level, and how this governs the outcome of infection and the physiological function of brain structures and cell types. This includes information on how the responses and cell composition develop dynamically over time. In this work, we have used temporally resolved RNA sequencing of single cells from HSV-1-infected mouse brains to address these questions.

## Materials and methods

### Mice

C57BL/6J mice were bred at Janiver-Labs. Isoflurane (Abbott), or [Ketamine (MSD Animal Health) + Xylazin (RompunVet)] was used to anaesthetize mice. Prior to experiments, mice were kept at the animal facility Faculty of Health Science, University of Aarhus. All mice used in this study were age-matched (7–12 weeks of age) male mice. All efforts were made to minimize suffering, and mice were monitored daily during infection.

### Viruses and reagents

Dulbecco’s Modified Eagle Medium (DMEM) was obtained from BioWhittaker and supplemented with 100 IU mL^− 1^ penicillin, 100 mg mL^− 1^ streptomycin and LPS-free FCS (BioWhittaker). HSV-1 (McKrae strain) was grown in Vero cells. The Vero cells used were from the lab stock. The titres of the stocks used were 5–10 × 10^9^ PFU mL^− 1^. Titres were determined by plaque assay on Vero cells. Beriglobin (CSL Behring) was used to neutralize extracellular HSV-1 and fully neutralized the virus in the dilutions used to calculate titres.

### Murine ocular HSV-1 infection model

Mice were anaesthetized with intraperitoneal (i.p.) injection of Ketamine (100 mg kg^− 1^ body weight) and xylazine (10 mg kg ^− 1^ body weight). Corneas were scarified in a 10 × 10 crosshatch pattern and mice were either inoculated with 2 × 10^6^ PFU HSV-1 in 5 µL of infection medium (DMEM containing 200 IU mL ^− 1^ penicillin and 200 µg mL^− 1^ streptomycin), or mock infected with 5 µL of infection medium. Mice were killed at the specified times post infection. Daily monitoring of weight and disease symptoms was performed during the infection period. Disease scores were determined as follows: head swelling (0, no bump; 1, minor bump; 2, moderate bump; 3, large bump. Mice that reached the humane endpoints or weight loss equal to or higher than 20% of initial weight were euthanized.

### Virus plaque assay

Brain stem was homogenized in a 2 mL Eppendorf tube by adding 500 mL of Dulbecco’s phosphate-buffered saline (DPBS) (Sigma-Aldrich) and a stainless-steel metallic bead (Qiagen) per sample in Tissuelyser (II) (Qiagen) for 5 min at a frequency of 50 Hz. The homogenized isolated tissue was centrifuged, and the supernatant was used for the plaque assay. Vero cells seeded in 6-well plates were inoculated with 100 mL of serially diluted samples and 900 mL of DMEM with 2% FBS. After 1 h of incubation, DMEM with 2% FBS and 0.4% human immunoglobulin were added to each well to neutralize any extracellular HSV-1. The plates were subsequently incubated for 2 to 3 days until virus plaques were visible and not overlapping. Staining with 0.03% methylene blue (laboratory stock) was used to visualize and allow quantification of the plaques.

### GeoMx

#### Tissue specimens

The mice were perfused, and the dissected brains were fixed with 4% formaldehyde, and then embedded in paraffin blocks. Tissue microarrays (TMA) were made from bregma − 5,52 to -7,92 containing regions of interest containing the brain stem and cerebellum, and by extracting 2-mm tissue cores from the paraffin blocks, followed by re-embedding these specimens into a single TMA block consisting of 43 cores in total from 15 mice. Cores without HSV-1 staining in the adjacent section were excluded resulting in 45 ROI from 9 infected and 3 control mice. The GeoMx DSP has the constraint of only being able to scan a slide area of 36,6 × 14,6 mm, so the TMA block was constructed such that the cores were confined to this area. Sections of 5 μm thickness were obtained from the TMA and were positioned according to the manufacturer’s instructions.

#### Tissue Preparation

The mounted sections were processed according to the manufacturer’s protocol (Nanostring Technologies MAN-10150-02). Sections were baked at 37 °C overnight followed by 60 °C for 1 h. Deparaffinization was performed by incubation in Xylene (VWR cat. nr. 28975.360) three times for 5 min. each. Rehydration was performed with 100% ethanol (EtOH) twice for 5 min. each, 95% EtOH once for 5 min., 70% EtOH once for 5 min., and 1X phosphate buffered saline (PBS) (BioNordika cat. nr. BN-53100) once for 1 min. Antigen retrieval was performed by submerging the slide into a 1X Tris-EDTA solution (Invitrogen, eBioscience cat. nr. 00-4956-58), at 80 °C for 15 min., followed by a wash in 1X PBS for 5 min. Target retrieval was performed by submerging the slide into a solution containing 1X PBS and 0.1 µg/mL proteinase K (Invitrogen cat. nr. AM2546) at 37 °C for 15 min., followed by a wash in 1X PBS for 5 min. Sections were postfixed in 10% neutral buffered formalin (NBF) (Sigma-Alrich cat. nr. HT501128), followed by two washes in a NBF stop buffer solution containing 2.42 g Tris base (Sigma-Aldrich cat. nr. T1503), 1.5 g Glycine (Sigma-Aldrich cat. nr. G7126), and 200 mL nuclease-free water (BioNordika cat. nr. BN-51100), for 5 min. each, followed by a wash in 1X PBS for 5 min.

Hybridization of photocleavable DSP RNA detection probes was performed by applying 220 µL hybridization solution to the tissue sections. The hybridization solution contained 25 µL DSP RNA whole transcriptome detection probes (GeoMx NGS RNA WTA Ms, Nanostring Technologies cat. nr. 121401103, which did not include probes for HSV), 25 µL nuclease-free water, and 200 µL Buffer R (GeoMx RNA Slide Prep Kit for FFPE, Nanostring Technologies cat. nr. 121300313). The slide was then covered with a hybridisation coverslip and incubated at 37 °C overnight. The following day the slide was dipped into a 2X saline sodium citrate solution (SSC) (Rockland Immunochemicals cat. nr. ROCKMB-045) to remove the coverslip, followed by two washes in stringent wash solution containing 4X SSC and 100% formamide (APPLICHEM cat. nr. 11237117), at 37 °C for 25 min. each, followed by two washes in 2X SSC for 2 min. each. Sections were then blocked with Buffer W (GeoMx RNA Slide Prep Kit for FFPE, Nanostring Technologies cat. nr. 121300313) and incubated at room temperature for 30 min. The TMA slide was stained with fluorescent-labelled primary monoclonal rat anti-mouse CD45-APC (BioLegend 30-F11, cat. nr. 103112) for 2 h. The antibodies were diluted in Buffer W at 1:20. After incubation the slide was washed in 2X SSC twice for 5 min. each. DNA was counterstained with 400 nM SYTO 83 (Thermo Fisher cat. nr. S11364) and incubated at room temperature for 1 h, followed by two washes in 2X SSC for 5 min. each. The slide was stored overnight in 2X SCC at 4 °C.

#### ROI selection strategy

The slide was loaded onto the GeoMx DSP instrument and scanned to produce a digital image of the tissue sections. The digital image was used to visualize tissue morphology based on the fluorescent labelled antibodies CD45 and the DNA stain SYTO 83. Regions of interest (ROI) were selected based on high morphological expression of immune cell-rich areas with a high expression of CD45, and SYTO 83. HSV-1 antibody staining was done on a separate sequential section as described. Multiple ROIs of various sizes (up to maximum ROI size of 660 × 785 μm) and with varying cell counts were selected.

#### ROI segmentation/aoi profiling and AOI collection

After ROIs were selected, the image analysis software integrated in the GeoMx DSP instrument was used to carry out threshold-based segmentation, to further divide each ROI into different segments. Segmentation into individual biological compartments was based on tissue morphology, CD45 expression, and cell count. Each ROI was divided into two segments: a segment containing individual cell populations with high CD45 expression, and a second segment containing cells with a positive nuclei stain but negative for CD45. Each segment within a ROI corresponds to one area of illumination (AOI). After segmentation of all ROIs into segments/AOIs, each AOI was exposed to ultraviolet (UV) light from the programmable digital micromirror device (DMD) built into the GeoMx DSP instrument. Each AOI was exposed to UV light in order to cleave DSP barcode tags from the AOI in question, releasing the tags from the tissue section. Released tags were collected by an automated microcapillary and dispensed into a 96-well microplate. After collection of all AOIs the microplate was sealed and stored at -20 °C.

#### Library preparation and sequencing

Library preparation was carried out according to the manufacturer’s protocol (Nanostring Technologies MAN-10153-03). The collected DSP tag aspirate was thawed and dried on a thermocycler at 65 °C, followed by resuspension in 10 µL nuclease-free water. A 4 µL aliquot of each resuspended aspirate was amplified in a PCR reaction containing NanoString SeqCode primers and PCR Master Mix (GeoMx Seq Code Pack_EF, NanoString cat. nr. 121400203). No probes for detecting viral genes were used. PCR products were pooled and then purified with two rounds of AMPure XP beads (Beckman Coulter cat. nr. A63882). Libraries were quality controlled using a 4200 Tapestation (Agilent) and QuBit v2 (Fischer Scientific), before being sequenced on an NovaSeq 6000 (Illumina).

#### Data analysis

The generated FASTQ files were converted to output DCC files using the GeoMx NGS Pipeline v2.5.1. In brief, raw reads were trimmed to remove adapter sequences. Overlapping paired-end reads were merged, and the resulting stitched reads were aligned to barcodes in the reference assay, hence creating aligned reads. The aligned reads were used to assign raw counts to biological target names. Reads matching the same barcode (PCR duplicates) were removed using the UMI region of each read, resulting in deduplicated reads converted to digital counts. Probe level counts were converted to gene level counts with the R package GeomxTools (v3.0.1). A limit of quantification (LOQ) for each segment was calculated as the geometric mean times the square of the geometric standard deviation of a set of negative probes. This LOQ was used as a segment specific gene filtering threshold, so that genes below LOQ in more than 2% of segments were filtered.

Transcriptional counts from each probe were imported, with genes retained only if at least one sample exhibited a read count above 5. Counts were subsequently normalized to Transcripts Per Million (TPM) and categorized into six groups based on brain region and CD45 selection criteria.

Gene expression was subsequently upper quantile normalized. Differentially expressed genes were calculated using linear mixed models (package lmer4 v1.1) with mouse individual as a random factor. The groups (CD45-neg cells in infected mice versus all cells Mock ROI) and (all cells in infected and all cells in mock ROI) were compared (Table S1). The biostatistical analysis of the differential expression matrices were conducted using the R Project for Statistical Computing(v4.3.3) and computed on GenomeDK. A gene set enrichment analysis (GSEA) was performed using clusterProfiler(v4.10.0) by applying the genome wide annotation for mouse(v3.18.0). For statistical significance of GSEA results a cutoff value of < 0.05 was used for observed enrichments of gene sets. Enriched terms of interest were selected for further analysis using gene-concept network visualization to identify active genes, their relative regulation, and statistical significance of regulation using DOSE(v3.28.1).

To manage computational demands when conducting deconvolution analysis for GeoMx data, the single-cell count matrix, initially comprising 143,915 cells, was down-sampled to a maximum of 5,000 cells per cell type, resulting in a total of 54,391 cells. This subset was then utilized to construct the signature matrix. The Dampened Weighted Least Squares (DWLS) method was applied for signature matrix construction [41]. Finally, deconvolution was performed using the DWLS method to calculate the predicted presentation score for each cell type within the grouped samples.

### Evaluation of BBB permeability

A 2% solution of Evans Blue in normal saline (4 mL/kg of body weight) was injected intraperitoneally. The stain was allowed to circulate for 4 h. Afterwards, the mice were transcardially perfused with 25 mL of PBS and fixed in 4% formaldehyde. The brainstems were dissected into 5-mm coronal sections. Evans blue dye leakage in mice brains from coronal and ventral positions was imaged on Leica M165FC microscope.

### Tissue immunofluorescence staining and immunohistochemistry

The brains were sectioned in the posterior-anterior direction on a freezing microtome (Brock and Michelsen, Thermo Fisher Scientific) into 40-µm thick coronal sections and separated into a series of ten. Sections were stored at -20 °C in an anti-freeze solution until use.

Immunohistochemistry was performed on free-floating sections. During the staining process, sections were washed multiple times in potassium-phosphate buffer (KPBS) between each incubation period. All incubation periods contained 0.25% Triton X-100 in KPBS. The sections were quenched for 10 min in a solution of 3% hydrogen peroxide and 10% methanol, followed by one hour of blocking with the appropriate 5% serum. The sections were incubated overnight at room temperature with the first primary antibodies monoclonal anti–HSV-1 (1/500, VP5 Abcam, clone 3B6), or anti-mouse CD3ε Antibody (1/500 Biolegend, clone 145-2C11) in 2.5% serum. The next day, sections were pre-blocked for 10 min in 1% serum before 2 h of incubation with the biotinylated secondary antibody in 1% serum, (1/200, anti-mouse, pre-absorption performed in mouse tissue before adding to experimental tissue), followed by 1-hour incubation with avidin-biotin-peroxidase complex (Vectorstain, ABCkit, Vector Laboratories) in KPBS. Development was done with 3,3-diaminobenzidine (DAB) and 0.04% Nickel. After the development of the first staining for HSV-1, the sections were washed and the above-described processes were repeated on the same sections for staining with rabbit anti-Iba1 antibody (1:700, Wako Fujifilm, 019-19741). Iba1 staining was developed with DAB but no Nickel. Sections were mounted on gelatin-coated glass slides and coverslipped.

For the adjacent sections to those used for GeoMx, the section mounted on glass was stained in a similar manner as described above and previously [24], using the rabbit polyclonal antibody anti-HSV-1 (1:500; Dako Cytomation, B0116).

### Quantification of Iba1 + cells in tissue sections

Two coronal sections containing the virus were identified per animal (mock, *n* = 3; day, *n* = 4; day 8, *n* = 5) (From bregma, Sect. “Background”: -7.48 to -7.76, Sect. 2: -7.20 to -6.84). Each hemisphere of the sections was divided into three concentrical areas starting from the edged of the tissue: 0–500 μm, which contained the most virus (Zone 0), 500–1000 μm (Zone 1), and 1000–1500 μm (zone 2) away from the area containing the most virus. In each of the 500–1000 μm and 1000–1500 μm areas, two photos (0.5 mm x 0.5 mm) were taken per section in both hemispheres. Extended focus imaging (EFI) photos were taken using an Olympus VS120 upright widefield slice scanner and the 40x objective. The z-range was set to 30 μm and the z-spacing to 1 μm.

Each photo was analysed in Fiji. Images were converted to 8-bit, and brightness/contrast was adjusted by the auto-function. The cell nuclei were counted using the multi-tool. The threshold was adjusted using the default setting. The infection was not equally effective in both sides of the brain and each animal is represented by two data points: one for the left and one for the right hemisphere. A two-way ANOVA with matched values followed by Tukey’s multiple comparison test was applied.

Microglia morphology was evaluated in the previously described acquired photos from Sect. 1 of each animal. Two photos from both zone 1 and zone 2 in both hemispheres were analyzed. The morphology of Iba1 + cells was determined by the length, thickness, number of processes, and characteristics of the cell body. Four profiles were defined: Type A (surveillant); no visible cytoplasm, small round dense nucleus, and long thin processes with little secondary branching. Type B (hyper-ramified); small but visible cytoplasm, round dense nucleus, and long processes with secondary branches. Type C (hypertrophic): Elongated and irregular cell body with visible cytoplasm, enlarged and less defined nucleus, shorter processes with varying thickness and less branching than type B. Type D (Ameboid); Big cell body merging with the processes, the nucleus fills out most of the cell body, and the processes are very short, thick and few. The average percentage of type A. B, C, and D of the total number of Iba1 + cells was calculated. Two-way ANOVA followed by Sidak’s multiple comparisons was applied.

### Preparation of single-cell suspensions of brain cells

Brain stems were harvested from animals sedated with ketamine and xylazine and thoroughly perfused with a minimum of 25 mL of PBS. Brain stems were cut into pieces and digested for 40 min at 37 °C with 5% CO_2_ in PBS supplemented with 1.6 IU mL^− 1^ Collagenase/Dispase (Roche) and 0.5 mg mL^− 1^ DNase I (Sigma-Aldrich). Twenty µL of sterile EDTA was added to each sample and incubated at 37 °C for another 5 min. Tissues were then mechanically disrupted through a 70-µm cell strainer into a single-cell suspension. Single-cell suspensions were washed with 25 mL 2% FCS-HBSS (Thermo Fisher Scientific), centrifuged, and resuspended in a mixture of PBS with 0.5% BSA (VWR) and Debris Removal Solution (Miltenyi Biotec). After overlaying PBS with 0.5% BSA, suspensions were centrifuged, resuspended, and adjust the cell count to 10^6^ cells mL^− 1^. We conducted standard Live/Dead using Propidium iodide (PI) and Hoechst. Cell Staining followed by flow cytometry analysis (Quanteon Q1), revealed high cell viability (> 80%).

### Single-cell RNA-sequencing preparation

Single-cell suspensions were converted into barcoded scRNAseq libraries following the manufacturer’s instructions of Chromium Single Cell 3’ Library, Gel bead & index kit and Chip G Kit (v3.1, 10x Genomics), aiming to recover 10,000 cells per library. Quality control of libraries was performed with Tapestation 4200. After QC, libraries were sequenced on an MGI DNBSEQ-G400 sequencer.

### scRNA-seq data pre-processing

Reads demultiplexed from the DNBSEQ-G400 were mapped to a composite reference genome consisting of the mouse genome (GRCm39) and the HSV-1 genome (JX142173) using Cell Ranger v6.1 from 10x Genomics. Predicted doublets identified by the Python package solo (v1.0) were removed before transferring the unique molecular identifier (UMI) count matrix into a Seurat object using the R library Seurat (4.4.0) for downstream analysis. Cells with more than 200 detected genes and less than 30% mitochondrial gene content were considered qualified and retained. After quality control, the dataset comprised 143,915 cells and 29,390 features. Table S2 contains quality control and viral reads. Normalization was performed using sctransform (v2) on all samples merged by conditions. CCA integration was employed for batch effect correction to ensure consistency across batches. Dimensionality reduction using PCA and UMAP, followed by clustering with KNN, was sequentially performed on the integrated count matrix to simplify and visualize the dataset and to identify distinct cell clusters.

Differentially expressed genes (DEGs) across various conditions, cell types, and cell subpopulations were identified using the FindMarkers function after reverting SCT assays to corrected counts with the PrepSCTFindMarkers function.

### Genomic copy number inference

The genomic copy number in cells with and without viral transcripts was estimated using single-cell transcriptional profiles analyzed by CopyKAT (v1.1). CopyKAT utilizes depth information from the gene expression levels of adjacent genes to infer the genomic copy number in the corresponding region. The analysis was conducted using CopyKAT’s default settings, requiring a minimum of five genes per chromosome to calculate DNA copy numbers. The segmentation parameter was set to 0.1.

### Cell type proportions analysis

To assess the dynamic changes in cell type proportions under various conditions, we employed statistical analyses to detect differences in cell type composition, which was conducted using the speckle (v0.0.3) which uses generalized linear mixed models to model cell counts and performs permutation tests to assess the significance of observed differences in cell type proportions. All proportion analyses were performed using the default settings of the speckle package. Cell population identities, conditions, and counts were extracted from the metadata of the Seurat object. Proportions for each cell population were initially calculated and then transformed using a logit function. An ANOVA was conducted to assess differences in cell population compositions across conditions. For each cell population, p-values and false discovery rates were calculated to determine if the proportions differed significantly among groups.

### Functional gene modules analysis

All identified DEGs ranked by fold changes were inputted to GSEA. DEGs with an adjusted p-value of ≤ 0.01 categorized as either upregulated or downregulated, were used for over-representation analysis against annotated gene sets. Gene Ontology (GO) enrichment analysis was conducted using R package clusterProfiler (v4.6.0). GO terms with q values of ≤ 0.01 were selected, and the enrichment value was defined as the ratio of genes in the category being considered to those in the background. Additionally, Disease Ontology analysis was performed using DOSE on human gene ortholog sets derived from mouse DEGs which were mapped via the babelgene (v22.9) R package.

Analysis of brain disease-related gene modules cited from BrainBase - CNCB-NGDC was conducted using SCPA (v1.5.4). SCPA assesses pathway activity by examining changes in the multivariate distribution of a given pathway across different conditions. Pathway differences were calculated for every subpopulation by setting the rest as the control populations.

### Subclustering analysis

Subclustering was conducted using MultiK (v0.1.0) and SigClust to determine the optimal number of clusters, allowing for a more precise identification of distinct cell subpopulations. MultiK was utilized to evaluate the stability and consistency of the clusters across multiple resolutions (from 0.05 to 2.00 with a step size of 0.05). MultiK allowed us to assess cluster robustness and select the most stable clustering configuration. SigClust was used to statistically test the significance of the identified clusters, ensuring that they represented true biological variation rather than random noise. We conducted subsampling and consensus clustering over 100 replicates to generate output for selection. Then MultiK diagnostic plots were plotted and used to determine the optimal K for clustering. Finally, diagnostic plots are made including dendrograms of cluster centroids with the pairwise SigClust p values mapped on the nodes and heatmaps of the pairwise SigClust p values for evaluation of the clustering results.

### Condition distribution preference score

To evaluate the tissue specificity of each subpopulation, we employed a methodology based on the previous study Pan-cancer single-cell landscape of tumor-infiltrating T cells (Liangtao Zheng et al. (2021). This approach involved analyzing the distribution of each subpopulation across different types of tissue and comparing the observed cell counts to those expected by random distribution. Specifically, we computed the ratio of observed to expected cell numbers (Ro/e) for each subpopulation under various conditions. By calculating the Ro/e ratios, we identified subpopulations that were enriched or depleted in specific conditions. This analysis provided insights into the condition-specific roles and potential functional implications of each subpopulation in the context of viral infection.

### Comprehensive analysis of cellular development pathways

We employed an integrated analytical framework to investigate cellular trajectories and gene regulatory dynamics within subpopulations. This approach combined Monocle3 (v1), Slingshot, RNA velocity analysis, Partition-based Graph Abstraction (PAGA), and SCENIC (v1.3.1) to comprehensively reconstruct lineage relationships and infer transcriptional regulation.

For monocyte subpopulation trajectory inference, we first identified subclusters and applied Slingshot by dyno to reconstruct lineage relationships and infer pseudotime, capturing the global structure of cellular transitions. RNA velocity analysis was then conducted, enabling us to determine the direction and pace of cellular transitions, as well as predict future cellular states based on transcriptional profiles. Furthermore, PAGA was utilized to generate a graph-based abstraction, offering a concise and informative representation of complex cellular transitions and interactions.

For oligodendrocyte developmental trajectory reconstruction, we employed Monocle3, while also applying RNA velocity and Slingshot to enhance the understanding and resolution of the inferred trajectories.

To gain insights into gene regulatory mechanisms, we utilized SCENIC, which identified gene regulatory networks and cell population-specific transcription factors with high regulon activity scores, shedding light on key regulatory drivers.

This integrated strategy allowed us to unravel dynamic cellular transitions and regulatory landscapes within monocyte and oligodendrocyte subpopulations.

### Cell-cell communication network analysis

The intercellular communication of literature-supported ligand-receptor interactions was inferred using CellChat (v1.5) among immune cells. The count matrix was imported to infer cell-cell communication probabilities at both the signaling pathway and gene levels. Selected significant signaling pathways and ligand-receptor pairs were identified and visualized using heatmaps and chord diagrams. Matrix factorization was applied to analyze outgoing and incoming communication patterns.

Differentially expressed and active ligand-receptor interactions between immune cells and blood-brain barrier (BBB) associated cells across different states were identified using the NicheNet and MultiNicheNet (v2.0.0) packages. NicheNet allowed us to pinpoint variations in cell-cell communication dynamics. To further elucidate the downstream effects, we inferred the signaling target genes of the communication pairs by assessing the enrichment of predicted target genes within the receiver cell types. The analysis was conducted with contrasts set among the conditions, using endothelial cells as receivers and either monocytes or other cell types as senders.

### Projection and mapping of query datasets

ScRNA-seq data was projected onto reference mouse TIL atlases using ProjecTILs (version 3.5). This approach facilitated a direct comparison within an annotated coordinate system, characterizing cell states that diverge from the reference.

The collected query datasets were mapped to our cells. This was achieved through cell type label transfer, which assigned subpopulation labels to the query cells. Subsequently, we projected these query cells onto reference UMAP embeddings using TransferData and MapQuery functions from Seurat package.

## Result

### HSV-1 CNS infection leads to influx of immune cells and disturbance of brain resident and vasculature cells

**Fig. 1:**
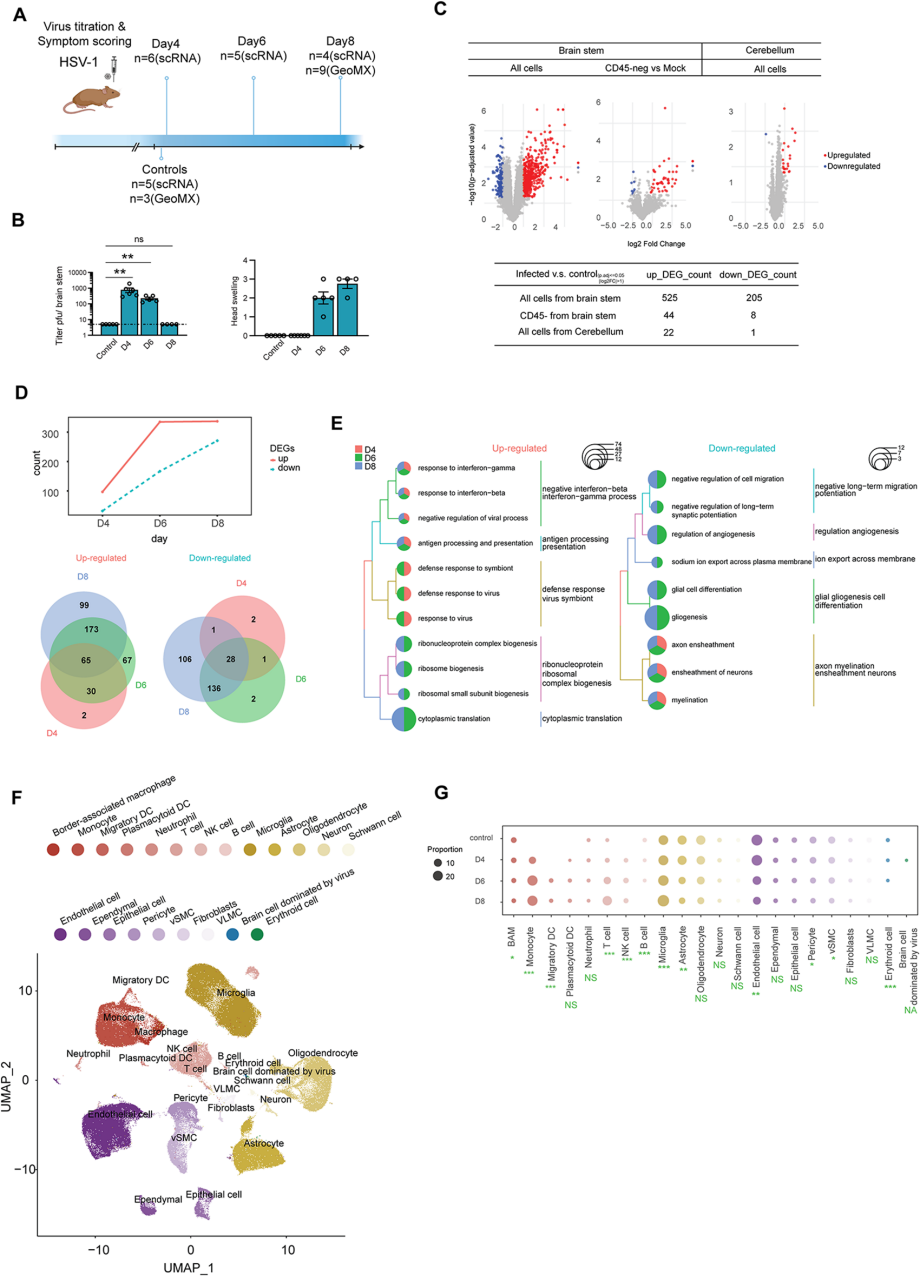
HSV-1 CNS infection leads to influx of immune cells and disturbance of composition and integrity of brain cells. (**A**) Experimental setup for single-cell and spatial transcripts profiling of mouse brainstem cells. (**B**) Bar plots illustrate the progression of viral titer and head swelling in the brainstem at various time points following viral infection. (**C**) Volcano plots showing differential gene expression between HSV-1-infected and control mice in GeoMx spatial profiles. The lower panel shows the count of upregulated and downregulated DEGs. (**D**) Number of differentially expressed genes in merged single-cell transcript profile from each time point after infection. Venn diagrams showing overlap of significant upregulated and downregulated genes between the time points. (**E**) Comparison of enriched GO terms of upregulated and downregulated genes from different time point after infection. The size of each pie presents the number of genes affected. (**F**) UMAP plot for dimensionality reduction and clustering from the sequenced mouse brain cells labeled with cell type annotation. (**G**) Bubble plot of cell type proportion; dot color represents cell type identity and dot size represents proportion. Data were analyzed using two-tailored one-way ANOVA. Asterisks (*) indicate *p* < 0.05, double asterisks (**) indicate *p* < 0.01, and triple asterisks (***) denote *p* < 0.001

To explore the dynamics of transcriptome changes in the HSV-1-infected brain, we inoculated C57BL/6 mice with HSV-1 in the cornea and isolated material on days 4, 6, and 8 for analysis (Fig. 1A). Infectious virus was detectable in the brain stem on day 4 and 6 post infection, and the mice developed signs of disease, which were observable on days 6 and 8. (Fig. 1B, Fig. S1A). To investigate the spatial transcriptomic changes in the microenvironments of the infected mouse brain, we employed GeoMx technology. Different regions of interest (ROIs) exhibiting signs of HSV-1 infection or infiltration of CD45 + cells in the brain stem or cerebellum were captured and sequenced. (Fig. S1B). This analysis revealed that alterations in gene expression were more pronounced in the brainstem compared to the cerebellum following HSV-1 infection, predominantly attributed to the presence of CD45 + immune cells (Fig. 1C).

Based on the above information, we employed single-cell RNA sequencing to analyze changes in the transcriptome profile of the mouse brain stem on days 4, 6, and 8 following HSV-1 infections. A first broad analysis of the changes of the host transcriptome showed that the fraction of reads mapping to antisense gene regions increased significantly on day 4 compared to the uninfected controls, followed by a decrease on subsequent days (Fig. S1D), also as seen previously in the context of HSV infections [42]. Second, the mRNA transcripts from cells in the infected brain stems contained progressive sequence mismatch to the C57BL/6 genome, indicative of expression of RNA-editing enzymes (Fig. S1E). Third, we identified top correlated expressed host genes with viral transcripts at each time point (Fig. S1F). *Il1rn*, encoding the IL-1 receptor antagonist, emerged as the top gene correlating between viral and host transcripts in single cells, and we identified myeloid cells to be the main source of Il1rn.

To prioritize the most significantly differentially expressed genes (DEGs) at each infection time point, we aggregated the replicates of single-cell transcript profiles within each time point into a consolidated transcriptome. Upon analysis of this dataset, we found that the number of upregulated genes exceeded the number of downregulated genes, and with a continuous increase in DEGs even beyond the time of presence of infectious virus (Fig. 1D, Table S3). When exploring the hierarchical clustering of enriched function of DEGs the upregulated DEGs were found to be enriched for immune functions, including IFN responses, antiviral activity, and antigen presentation (Fig. 1E). Among the downregulated DEGs, the enriched pathways included many functions related to physiological brain function, including gliogenesis, axon ensheathment and angiogenesis (Fig. 1E).

From the mice subjected to single-cell RNA sequencing, a total of 143,915 high-quality single cells were obtained. Our analysis revealed a comprehensive spectrum of 22 distinct cell types, encompassing 8 immune cell types, 5 brain resident cell types, 8 cell types involved in vasculature and tissue structure (Fig. 1F, Fig. S1G). Following HSV-1 infection, influx of monocytes, dendritic cells (DC), and NK cells were detected from day 4, with particularly the number of monocytes increasing and continuing through the time course of the experiment (Fig. 1G). When looking at the resident brain cells and cells associated with vasculature and tissue structure, we observed that microglia numbers dramatically increased to become the most abundant cell type identified in the samples from mice infected for 8 days. By contrast, we observed a profound and temporally progressing decrease in the number of astrocytes in the HSV-1-infected brain stem. A similar loss of markers defining endothelial cells and pericyte cells following infection was also observed. Endothelial cells and pericytes are all the key constituents of the BBB (Fig. 1G, Fig. S1H-I). This was not merely due to change in cell proportions, due to influx of immune cells, since analysis of the brain resident, vasculature, and tissue structure cells alone, also showed a reduction in absolute cell counts of markers defining astrocytes, endothelial cells, and pericytes (Fig. S1H). Collectively, these data demonstrate that the HSV-1-infected brain is subject to a transcriptional stress response indicated by reduced transcriptional integrity, elevated immune activity, and down-modulation of homeostatic brain pathways. This is paralleled by a profound increase in microglia, influx of monocytes, and loss of specific cell types, notably astrocytes, pericytes, and endothelial cells.

### Alterations in transcriptional profile following HSV-1 infection

**Fig. 2:**
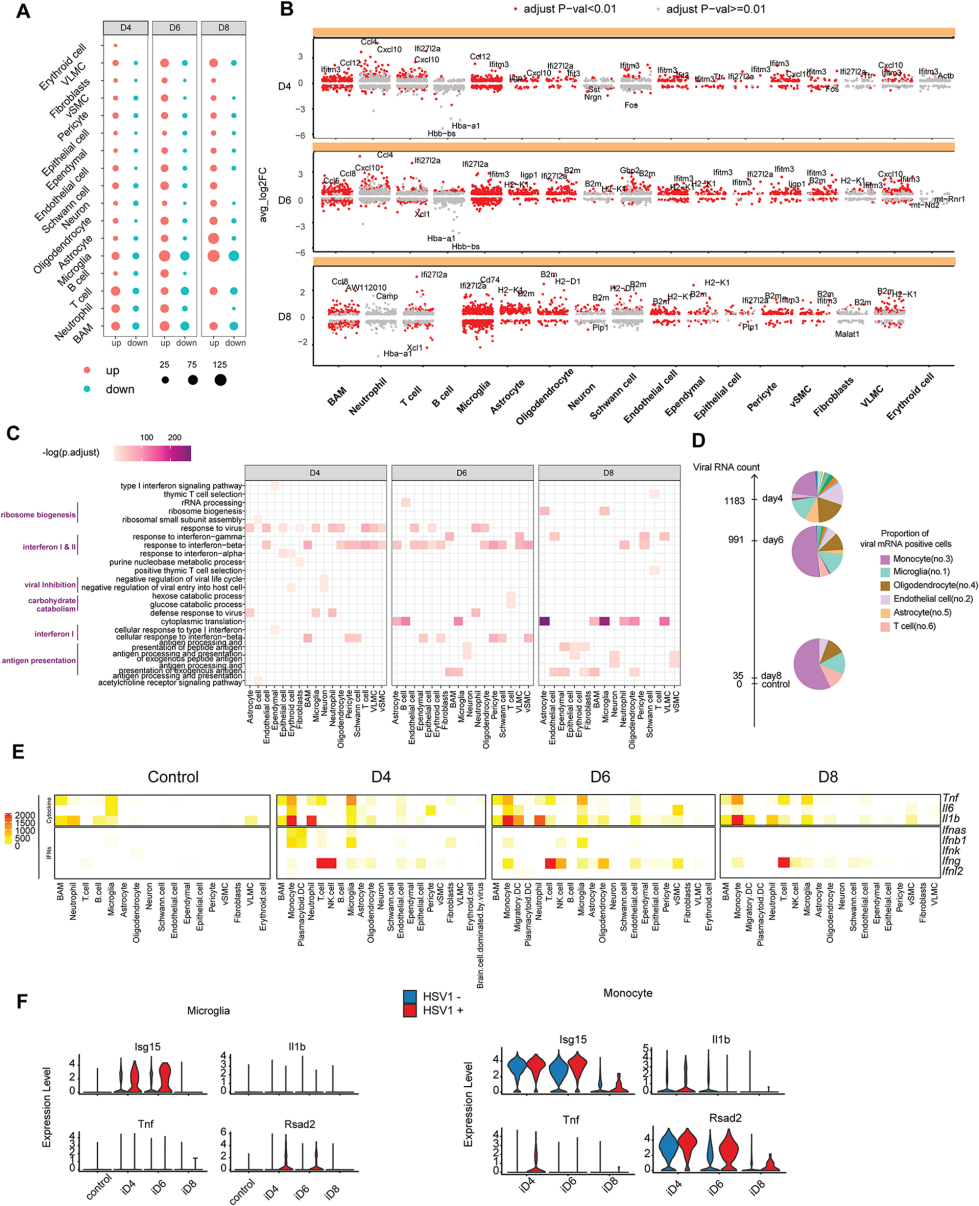
Alterations in transcriptional profile following HSV-1 infection. (**A**) Bubble plot of number of differentially expressed gene in identified cell types compared with control samples. (**B**) Scatter plots displaying differentially expressed genes compared with controls, with significantly differentially expressed genes highlighted by red dots. Only top two significantly differentially expressed genes are labeled. Differential expression testing is conducted using the non-parametric Wilcoxon rank-sum test. (**C**) Heatmap showing top two enriched functions categorized into interferons responses, antiviral activity, and antigen presentation across various cell types at different infection time points. (**D**) Pie charts illustrating the distribution of viral transcript loads across various cell types on days 4, 6, and 8. The numbers next to the axis represent the number of cells containing viral transcripts at each time point.The number following the cell type name indicates the rank of cell abundance. (**E**) Heatmap displaying the expression patterns of selected interferon and cytokine genes in the annotated cell type. (**F**) Expression of cytokines and ISGs in monocytes and microglia containing HSV-positive (HSV+) and HSV-negative (HSV-) cells under various infection conditions

Next, we analyzed the DEGs at a finer resolution, with the aim to pinpoint comprehensive alterations in transcriptional profiles in the different cell types following viral infection. In line with the general observation that more DEGs were upregulated than downregulated (Fig. 1D), this was also observed in most cell types (Fig. 2A). The microglia exhibited the highest number of DEGs, and upregulated genes included the chemokines *Ccl2*, *Ccl5*, and *Ccl12;* the IFN-stimulated genes (ISGs) *Ifi27l2a*, *Ifitm3*, and *Bst2*; and the MHC genes *H2-K1* and *H2-Ab1* (Fig. 2B, Table S3). Although of lower amplitude, most other cell types also showed early induction of many ISGs and chemokines, and later induction of antigen-presentation-related genes. This was confirmed in a pathway enrichment analysis (Fig. 2C, Fig. S2A-F). Of note, the above analysis did not include monocytes, the most abundantly recruited immune cells, since they could not be compared to control brains, which did not contain monocytes.

As the next part of the initial characterization, we first investigated the abundance of HSV-1 transcripts. Significant amounts of viral transcripts were observed on day 4 and 6 post infection (Fig. 2D), which was in line with the detection of infectious viral particles in the brain stem (Fig. 1B). Interestingly, viral transcripts were found to be particularly enriched in monocytes and microglia (Fig. 2D), which have low permissiveness for HSV-1 infection but have the ability to move within the brain [12, 42], suggesting uptake of virus and virus-infected cells. Second, we investigated which cell types that expressed the cytokines which are frequently involved in physiologically important for immune cascades. We noted that the type I IFN gene transcripts were mainly derived from microglia and monocytes, as well as the very low-abundant plasmacytoid dendritic cells, peaking on day 4, while a strong *Ifng* expression (type II IFN) was observed throughout the time of the infection from NK cells and T cells (Fig. 2E; Fig. S2G). With respect to inflammatory cytokines, we observed strong induction of notably *Tnfa* and *Il1b* by monocytes and microglia, with monocytes being the main source and extending beyond the duration of viral presence on the brain (Fig. 2E). To investigate whether virus-sensing cells or bystander cells expressed cytokines, we dissected the temporal cytokine induction in monocytes and microglia (Fig. 2F). For the IFN response, we observed that while microglia largely relied on presence of virus to evoke this response, monocytes did not. The inflammatory cytokines *Il1b* and *Tnf* were expressed to higher levels in monocytes than microglia with the *Tnf* response being strongest in HSV-1+ cells, whereas *Il1b* expression in the monocytes was equal between HSV-1 + and HSV-1 ÷ cells. This indicates that monocytes exhibit both anti-viral and pro-inflammatory responses upon entering the brain, regardless of virus detection, whereas resident microglia only respond upon sensing the virus. Collectively, these results show that the immune response in the HSV-1-infected brain is dominated by early IFN and antiviral responses and subsequent activation of antigen presentation, with most cell types being involved notably microglia and monocytes. Monocytes contribute substantially to the type I IFN genes and the prolonged expression of proinflammatory cytokines.

### Exploration of microglia activity and subpopulations

**Fig. 3:**
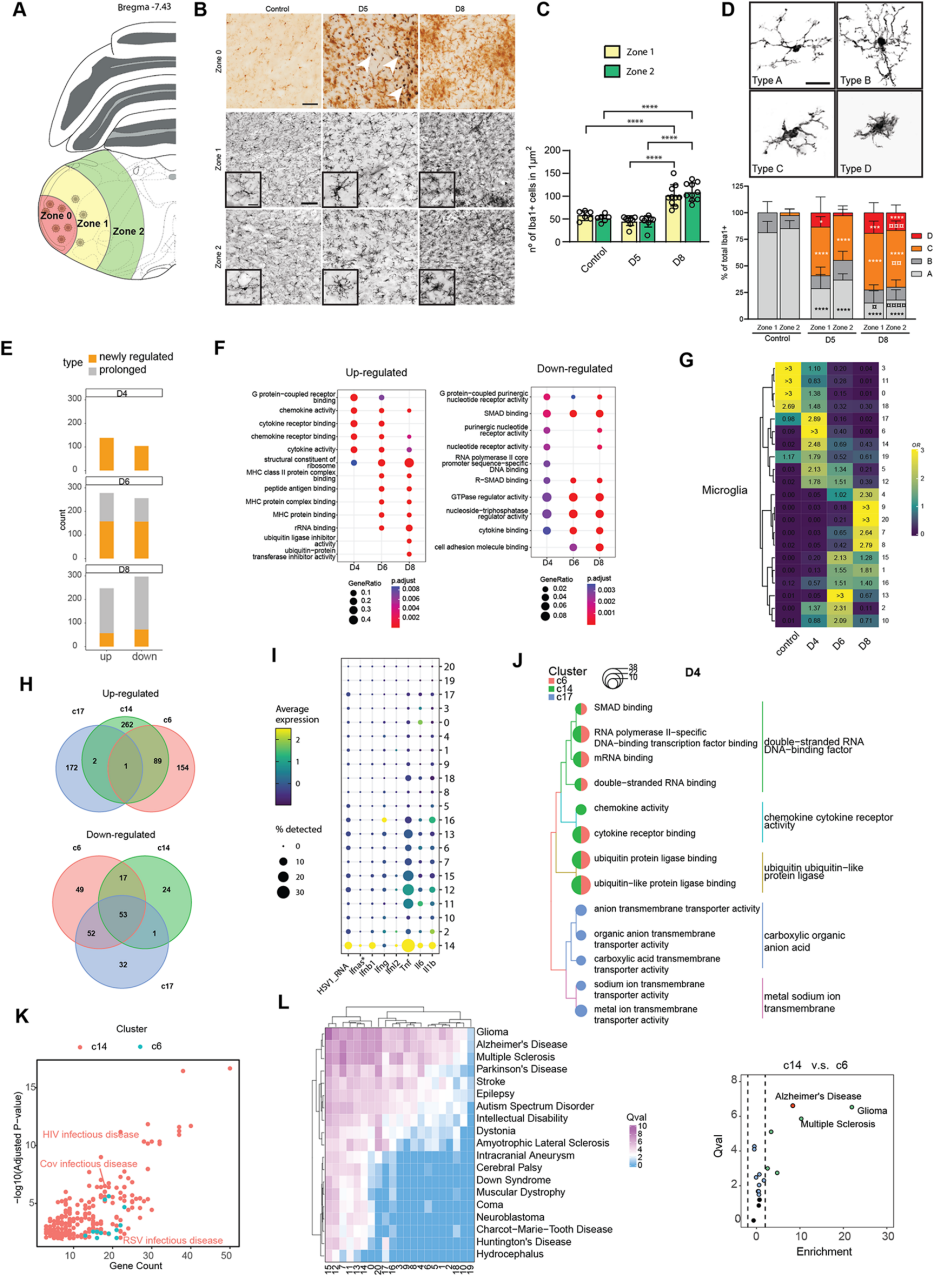
Transcriptional and functional heterogeneity within subpopulations of microglia. (**A**) Illustration of area in the brain stem, categorized as zones 0, 1 (500–1000 μm from center of virus focus), and 2 (1000–1500 μm from center of virus focus). (**B**) Representative images from double staining of brain stem sections with anti-HSV (black) and anti-Iba1+ (brown). Representative HSV + cells are shown by white arrows. Scale bar, 50 μm. Inserts display changes in the morphology of Iba1 + cells. Scale bar, 20 μm. (**C**) Number of Iba1 + cells were quantified for all three conditions and in zone 1 and 2. (**D**) Representative images of Iba1 + cells grouped in the different morphology categories A-D. Scale bar in 40x photos represents 50 μm. Percentage of Iba1 + cells in the different morphology classes were quantified in zone 1 and 2. (**E**) Bar plots showing the number of DEGs in microglia at various infection time points. Orange, newly regulated genes; gray, genes also differentially regulated at the previously examined time pointed (i.e. two days earlier). (**F**) Comparison of enriched functional terms for upregulated and downregulated DEGs in microglia. (**G**) Heatmap showing the prevalence of microglia subpopulations at different time points, estimated by the Ro/e score. The numbers on the left indicate the subpopulation’s rank, with lower numbers indicating higher abundance and higher numbers indicating lower abundance. (**H**) Venn diagram of DEGs in subpopulations #6, #14, and #17. (**I**) Dot plot of selected cytokine and interferon genes, along with HSV transcripts. (**J**) Comparison of enriched function term of upregulated genes in subpopulations #6, #14 and #17 on day 4 post infection. (**K**) Scatter plots of enriched human disease terms in subpopulations #6 and #14. (**L**) The left panel presents a heatmap of brain disease-related pathway activity values in microglia subpopulations. The right panel compares the pathway scores between subpopulations #6 and #14. Panel C, data are presented as ± SD. Two-way ANOVA with matched values, followed by Tukey’s multiple comparisons test was applied (*< *p* = 0.05 ****p ≤ 0.0001). Mock, *n* = 6 (left = 3, right = 3), D5, *n* = 8 (left = 4, right = 4), D8, *n* = 10 (left = 5, right = 5). Panel D: data are presented as + SD. Two-way ANOVA with matched values when analyzing zone 1 and zone 2 within the same group, or without matched values when analyzing the same zone across groups. * compared to the same zone in the mock (*< *p* = 0.05, ***p ≤ 0.001, ****p ≤ 0.0001). ¤ the same zone compared to 5 DPI (¤< *p* = 0.05, ¤¤*p* ≤ 0.01, ¤¤¤*p* ≤ 0.001, ¤¤¤¤*p* ≤ 0.0001). Mock, *n* = 6 (left = 3, right = 3), 5 DPI, *n* = 8 (left = 4, right = 4), 8 DPI, *n* = 10 (left = 5, right = 5)

Given the substantial effect of HSV-1 infection on the proportion and gene expression in microglia, we wanted to characterize this cell type in more detail. Using Iba1 immunostaining in differences radial distances from the infection foci, zone 0–2 (Fig. 3A), we observed an absence of the characteristic ramified microglia in the center of the infection foci (zone 0, Fig. 3B) and an increase in the number of cells in the periphery and adjacent areas (zone 1 & 2, Fig. 3B, C). Comparing the morphological profile of the microglia showed a profound shift from the ramified surveilling shape abundant in the mock brains (type A, Fig. 3D) towards the hypertrophic and amoeboid shape (types C and D) at day 5 following infection, indicating activation (Fig. 3D). It is important to note that the amoeboid microglia cannot be distinguished from infiltrating monocytes through the method used. The amoeboid microglia/monocytes (type D) were more abundant in zone 1 than in zone 2 on day 5 (14% vs. 3%), while they were equally abundant in the two zones on day 8 (20% and 17%), when the virus had been cleared. In fact, at this point, over 70% of the microglia in both zones showed the more activated profiles (type C and D) that were very rare in the homeostatic brain (Fig. 3B, D), suggesting an ongoing microglia response in areas distant from the infection foci even after the infection has been resolved.

To explore changes in the transcriptomic profiles of microglia upon viral infection, we compared the overall transcriptomic alterations at different infection time points and found significantly greater changes in the microglial transcriptomes on days 6 and 8 compared to day 4 post-infection (Fig. 3E). Genes significantly upregulated in microglial cells on days 4 and 6 are predominantly associated with cytokine activity. Conversely, on days 6 and 8, significantly upregulated genes were enriched in functions related to antigen-presentation and protein synthesis, the latter indicating cellular activity and proliferation, which is in consonance with the increased number of Iba1 + cells (Fig. 3C, F left, Fig. S3A). The genes downregulated in microglia were enriched in the pathways SMAD binding, cytokine binding, and cell adhesion molecules binding, which play critical roles in regulating microglial function, immune responses, and interactions within the CNS microenvironment (Fig. 3F right, Fig. S3B). Alteration of these signaling pathways may promote changes in actin cytoskeleton organization and interactions with neighboring cells, leading to morphological shifts in microglia.

To investigate the heterogeneity of microglia following viral infection, we analyzed subpopulations by further dividing them into distinct subclusters. Condition enrichment scores reveal the distribution of subpopulations at the infection time points post infection (Fig. 3G, Table S3). Importantly, there was a very dynamic change in the microglia population composition during HSV-1 brain infection. Further characterization of the subpopulations showed that the dominating subpopulations on day 4, subpopulations #6, #14, and #17, were transcriptionally rather distinct (Fig. 3H). Most notably, subpopulation #14 was the one containing most HSV-1 RNA and expressing the highest levels of transcripts for type I IFN genes and the inflammatory cytokines *Tnfa*,* Il1b*,* and Il6* (Fig. 3I). This subpopulation also expressed the highest levels of chemokine transcripts, and high levels of cell death-associated genes and nucleic acid-binding proteins (Fig. 3J; Fig. S3C-G). Importantly, subpopulation #14 was also associated with inflammation and disease phenotypes (Fig. S3H), including viral diseases, and neurodegenerative diseases (Fig. 3K, L). This subpopulation was also a key contributor to the downregulation of glial cell differentiation (Fig. S3I), thus further underscoring the dual nature of #14, particularly if present over longer times. Among the subpopulations that dominated on day 6 and 8, key activities were antigen presentation, proliferation, and repair (Fig. S3C-E). Subpopulations #15 and #16, which have high expression levels of *Top2a*,* Mki67*, and *Stmn1*, were classified as proliferating (Fig. S3H), and both were enriched in functions related to tubulin binding (Fig. S3D). These two subpopulations peak on day 6, with the former being distinguished by its enrichment in MHC protein binding function (Fig. S3D). Finally, within the subgroups identified as enriched on day 8, subpopulation #9 stands out for its particularly high expression of Lgals3, a marker gene associated with clearance repair function (Fig. S3H). In summary, HSV-1 infection leads to a profound change in the microglia phenotypes and includes the highly antiviral and proinflammatory subpopulation #14, which is present early and transiently in the HSV-1-infected brain microenvironment.

### Investigation of heterogeneity and differentiation within monocyte subpopulations

**Fig. 4:**
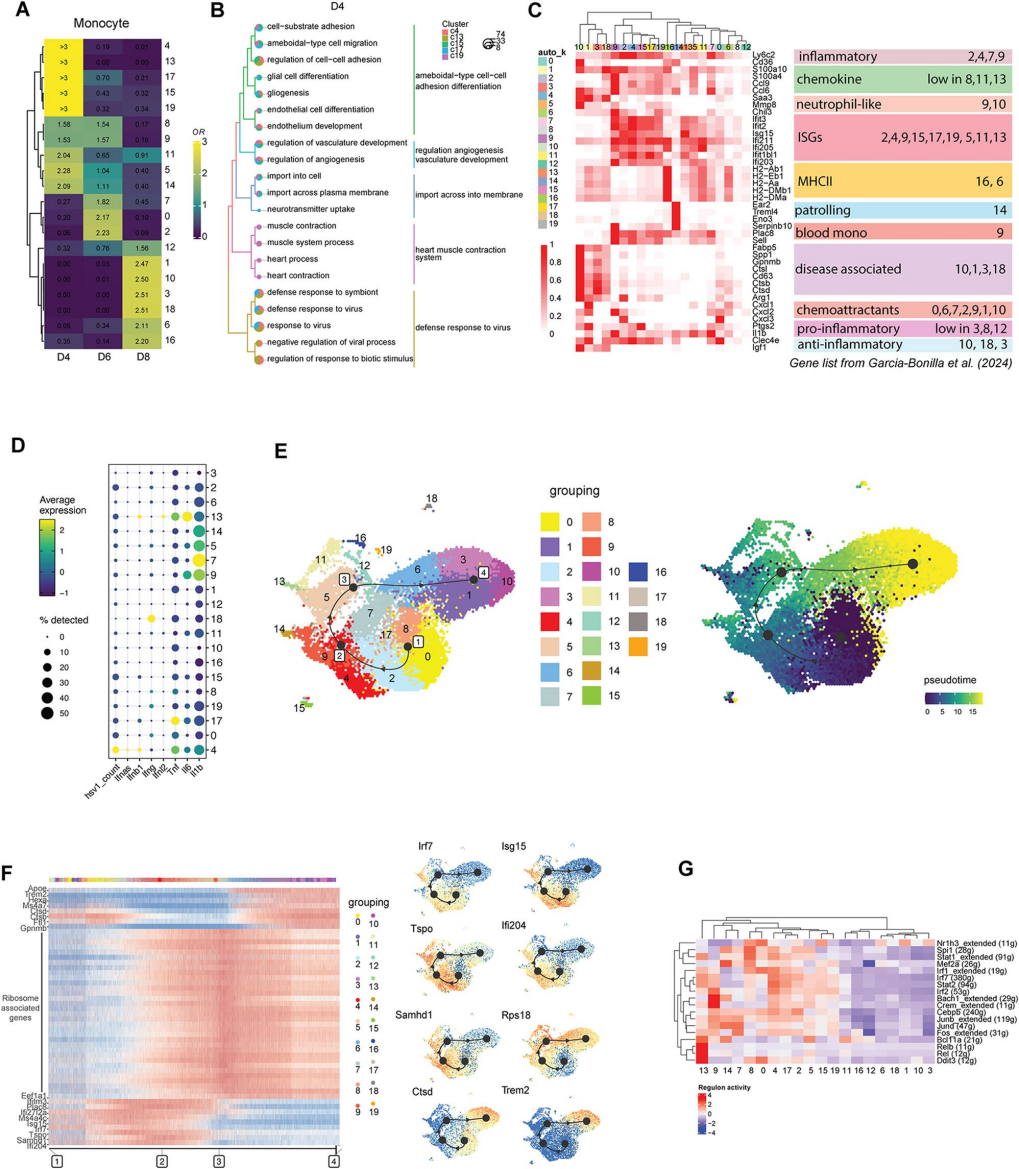
Heterogeneity monocyte subpopulations in the HSV-1-infected brain. (**A**) Heatmap showing the prevalence of monocyte subpopulations at different time points, estimated by the Ro/e score. The numbers on the left indicate the subpopulation’s rank, with lower numbers indicating higher abundance and higher numbers indicating lower abundance. (**B**) Enriched function terms in upregulated genes in subpopulations prevalent on day 4 post infection. (**C**) Heatmap of characteristic marker genes for monocyte subpopulations, including functional annotation of the groups as described [79]. (**D**) Dot plot of viral transcripts and selected cytokine and interferon genes. (**E**) Left: UMAP of the inferred trajectory with connected milestone points, labeled by subpopulation identity. Right: UMAP of the inferred trajectory colored by pseudotime order. (**F**) Heatmap overview of the most predictive genes along the inferred trajectory and selected feature plots. (**G**) Heatmap showing the regulon activity scores of subpopulation-specific transcription factors

Recruited monocytes progressively accumulated in the infected mouse brain stem, reaching a proportion of over 20% by day 8. Within the large population of monocytes, 20 subclusters were identified as exhibiting heterogeneous features revealed by distinct gene expression patterns of subsets (Fig. 4A, Fig. S4A-B, Table S3). Among the subpopulations abundant on day 4, particularly populations #4 and #13, as well as population #17, are characterized by ISG expression and antiviral responses (Fig. 4B, C). Additionally, these subpopulations were enriched for regulation of vasculature (Fig. 4B), likely contributing to the loss of BBB integrity. Among these, subpopulation #4 contained the highest level of viral RNA and was categorized as Ly6c2 positive inflammatory monocytes with expression of all the cytokines *Tnfa*, *Il1b*, and *Il6* (Fig. 4C, D, Fig. S4C). A small fraction of the cells from this subpopulation expressed high levels of type I IFN genes (Fig. 4D). By contrast, subpopulation #13 exclusively expressed high levels of *Il6*, and #17 predominantly expressed *Tnfa* (Fig. 4D).

As was also seen for the microglia subpopulations, the monocyte subpopulations showed a high degree of dynamics, and the most abundant populations on day 4 post infection were barely detectable on day 6 (Fig. 4A). The monocyte subpopulations dominating on day 6, #0, #2 and #7, were categorized as inflammatory and chemoattractant (Fig. 4C). The biological activities at this time point were characterized by a shift from pattern recognition receptor (PRR)-driven cytokine responses towards antigen-presentation and T cell activation, including IFN-γ responses (Fig. S4D). Among the monocyte subpopulations dominating on day 6, #7 stood out as being the main source of *Il1b* among all subpopulations (Fig. 4D). The transcriptome from the subpopulation showed enrichment of a range of proinflammatory activities, including regulation of leukocyte cell-cell adhesion and activation (Fig. S4E). Interestingly, subpopulation #7 did not contain significant levels of viral RNA (Fig. 4D), thus suggesting a more complex mode of induction than PRR stimulation alone. In this regard, we noted that this subpopulation also showed enrichment in IFN-γ responses, known to be a major driver of monocyte/macrophage activation (Fig. S4E). The strong downregulation of processes associated with chemotaxis and upregulation of processes involved in cell-cell adhesion suggests recent recruitment to the area (Fig. S4E). Finally, the monocyte functions enriched on day 8 post infection still included antigen presentation, but also more regulatory activities of immune responses (Fig. S4F). Among the monocyte subpopulations identified on day 8, subpopulations #10, and #18 expressed the C-type lectin Clec4e (MINCLE) (Fig. 4C). This protein has been reported to be a cellular sensor for necrosis [43], which is a signature of HSE [44], and may thus be central for clearance of debris from the HSV-1-infected brain. Additional functional enrichment analysis revealed that cluster #10 suppresses key inflammatory activities (Fig. S4G-H). In contrast, subpopulations #6 and #16 notably expressed MHC-II related genes, thus suggesting that the pro- and anti-inflammatory activities of infiltrating monocytes are mediated by distinct populations of cells at this stage of the host response to the infection. The observation that subpopulation #16 is undergoing cell division (Fig. S4F), could indicate a prolonged presence in the post-infectious brain.

Considering the prevalence at different time points and diverse antiviral functions, the differentiation trajectory of the recruited monocyte subpopulations was explored using comprehensive trajectory inference analysis (Fig. 4E, Fig. S4I-L). The sequence of subpopulations appearing according to the pseudotime was consistent with the findings in real-time (Fig. S4I). The significant variability in gene expression along the trajectory also revealed a transition of recruited monocytes from an antiviral stage to a tissue homeostatic stage (Fig. 4F). The IFN-regulated genes *Ifi204*, *Irf7*, and *Isg15*, were all highly expressed at the onset of the immune response trajectory. This overlapped with, and was exceeded by, the expression of the activation marker *Tspo*, which in turn was followed by the proliferation marker *Rps18*. *Trem2* was highly expressed at the end stage of the recruited monocytes. *Trem2* plays a crucial role in regulating inflammation, phagocytosis, and the maintenance of tissue homeostasis. The inferred transcription factor regulon based on the single-cell transcriptional profile revealed more intensive regulon activity at the initial stage of the monocyte trajectory compared to later stages. Specifically, IRF7 and STAT2 regulate gene expression in subpopulation #4, NF-κB subunits Rel and RelB play significant roles in subpopulation #13, while Jun-Fos family members contribute to the activity of subpopulation #7 (Fig. 4G). Collectively, the monocytes recruited into the HSV-1-infected brain show high functional diversity and dynamical change over time.

### Monocytes are key mediators of immune cell crosstalk and spatially related to the site of infection

**Fig. 5:**
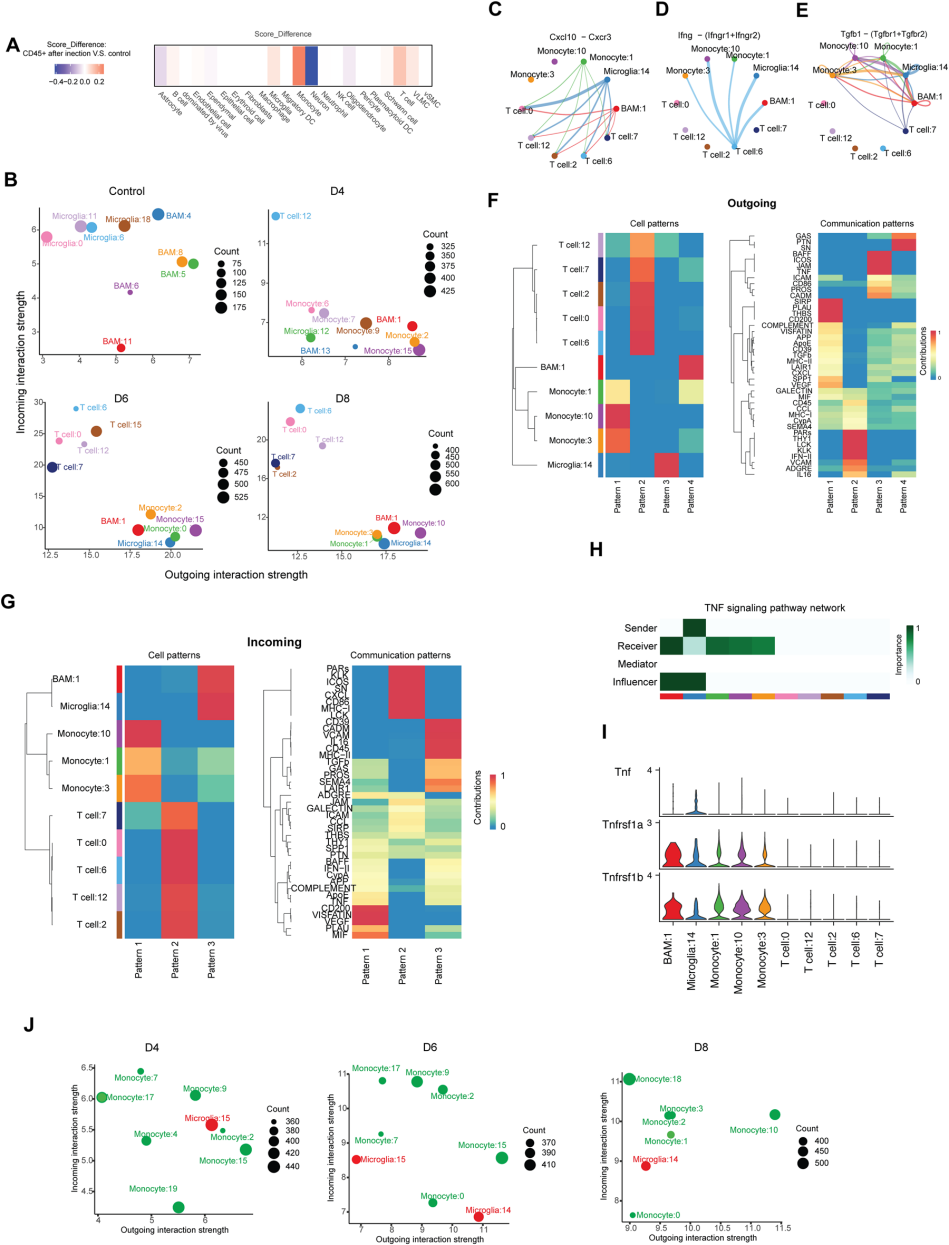
Monocytes are key mediators of immune cell crosstalk and spatially related to the site of infection. (**A**) Heatmap depicting deconvolution of GeoMx data. Score differences of various cell types between the CD45 + compartment of the infected brainstem and the control mice are shown. (**B**) Dot plot illustrating the top 5 dominant sender. (source) and receiver (target) subpopulations. Dot size corresponds to proportional to the number of inferred links associated with each subpopulation. (**C-E**) Aggregated cell-cell communication network representing the *Cxcl10*, *Ifng* and *Tgfb1* signaling sending from each cell group. The network is summarized by aggregating communication probabilities. (**F**) Outgoing communication pattern showing how these cell groups coordinate and how signaling pathways collaborate to send signals. (**G**) Incoming communication pattern indicates how these cell groups coordinate and how signaling pathways integrate to receive signals. (**H**) Heatmap displaying the network centrality scores of TNF signaling pathway. (**I**) Violin plots showing the TNF signaling genes expression pattern in the indicated subpopulations. (**J**) Dot plot illustrating the top five dominant sender and receiver subpopulations within inferred communication networks among microglia and monocyte subpopulations across different infection time points

Following the identification of monocyte populations in the HSV-1-infected brain stem, we wanted to examine whether they could be spatially linked to the infected brain region. Therefore, we deconvoluted the GeoMx RNA transcriptome from the region around the brainstem principal trigeminal nucleus, which is the primary site of HSV-1 infection in this model. Indeed, we identified monocytes in high abundance, but also microglia and T cells in the infected regions of the brain stem (Fig. 5A). Therefore, we looked in more detail into the possible crosstalk between immune cells in the infected brain stem, since this may contribute to amplification of both protective and pathological processes in the CNS. T cells showed a significant increase in proportion and emergence of Th1 cells, while border-associated macrophages (BAM) decreased proportionally (Fig. S5A-C).

When investigating the dynamics of immune cell interactions, we observed a shift from dominance of BAM-to-microglia crosstalk in the uninfected brain to monocytes-derived signaling to mainly T cells after HSV-1 infection (Fig. 5B). The monocytes showing the strongest communication with T cells included both inflammatory subsets #2, #7, and #9, MHC-expressing subset #6, and on day 8 also anti-inflammatory subsets #3 and #10. The top signal-receiving T cell subpopulations were #12, #7, #6, and #0, strongly emerging from day 6 to day 8 (Fig. 5B). When looking in more detail at the nature of cell-cell communication, we found numerous pathways to be affected (Fig. 5C-E; Fig. S5D-F). For instance, CXCL and CCL chemokine expression was a major player, likely directing the recruitment of immune cells into the CNS and to the site of infection/inflammation (Fig. 5C; Fig. S5D-F). *Cxcl16* was the dominant ligand, broadly expressed across the top five senders, while *Cxcl9* had a more restricted expression, found primarily in BAM #1, microglia #14, and monocytes #1 (Fig. S5F). Similarly, *Cxcl10* was detected in BAM #1 and microglia #14, with *Cxcl11* exclusively expressed in microglia #14. This highlights the pivotal role of microglia #14 in the early stages of immune response to HSV-1 infection. Although T cells were mainly identified as receivers of signals, we noted subpopulation #6 as a striking sender of IFN-γ signaling to a panel of monocyte populations (Fig. 5D). Moreover, TGFβ signaling between three major monocyte populations infection #1, #3, and #10 present on day 8 post, and also crosstalking with T cell #7 suggest this cytokine to be an important player in the post-infectious curbing of the proinflammatory response, to limit tissue damage (Fig. 5E).

We examined immune cell communication patterns more globally (Fig. 5F, G; Fig. S5G). This analysis revealed specific patterns of communications, suggesting coordinated conditioning of the infection microenvironment (Fig. 5F, G). For instance, TNF signaling was a major player in communication patterns identified on both days 4, 6, and 8 (Fig. 5F, G; Fig. S5G), and microglia cluster #14 was a key sender, with several monocyte subpopulations receiving the signal (Fig. 5H-I). Finally, we incorporated the temporal dimension of the monocyte-microglia communication pattern (Fig. 5J). This analysis clearly showed that more monocytes subpopulations than microglia subpopulations are actively engaged in ingoing and outgoing cell-cell communication, and this includes both monocyte populations that are highly positive and less positive for HSV-1 RNA. Among the microglia populations, #14 and #15 are engaged in communication with monocytes. These finding confirm the above data, suggesting that virus-sensing microglia communicate extensively with other cells, while monocytes communicates extensively with T cells, but also microglia, in a manner not primarily driven by response to direct sensing of virus. Altogether, these data suggest strong, coordinated, and dynamic communication between resident and recruited immune cells into the brain following HSV-1 infection.

### Transcriptional changes and cell-cell communication mediating disturbance of BBB integrity

**Fig. 6:**
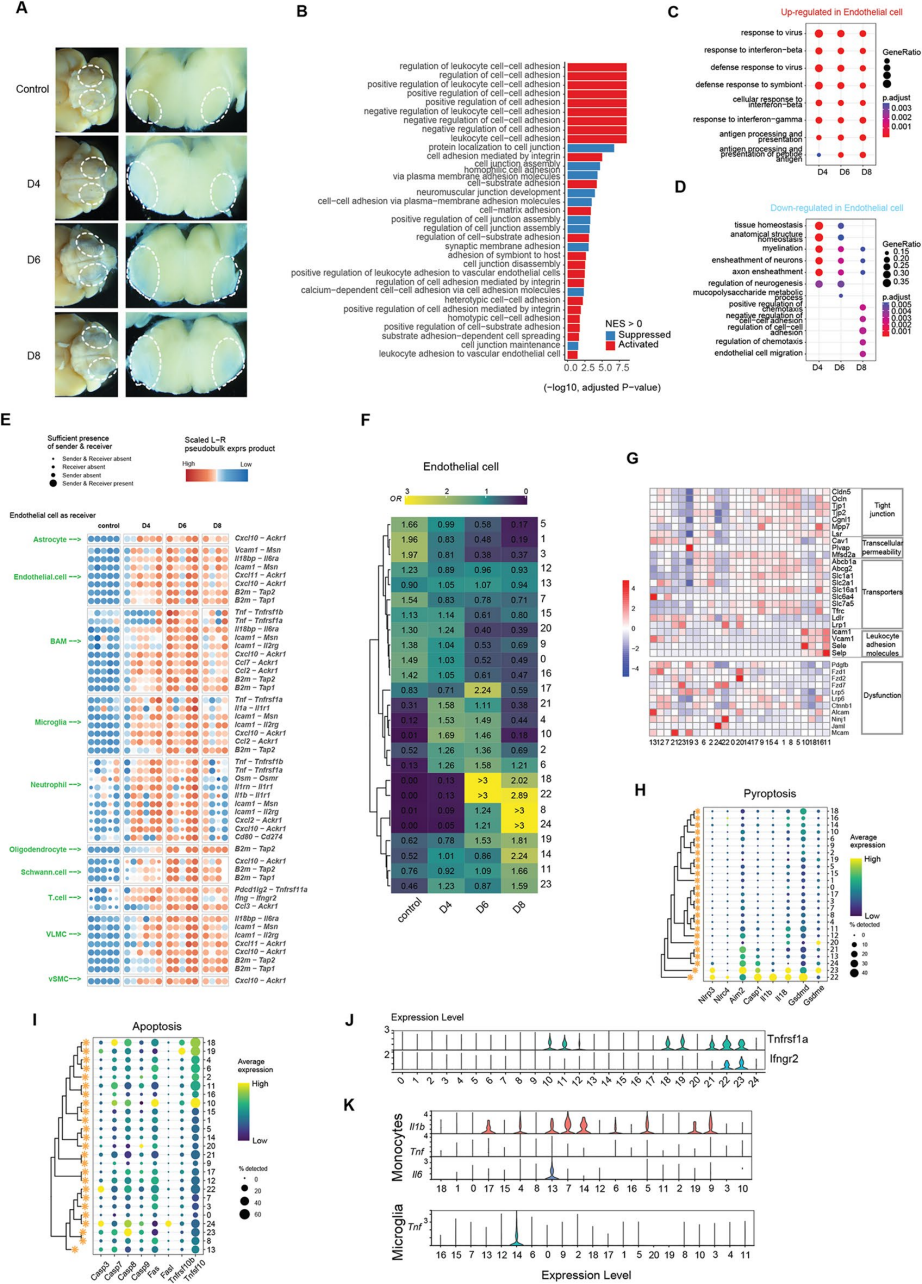
Transcriptional changes and dynamic communication in cells involved in the weakened blood-brain barrier post-infection. (**A**) Mock- and HSV-1-infected mice received Evans blue dye by the i.p. route 4 h before transcardial PBS perfusion. Representative images of Evans blue dye leakage (white ovals, indicates BBB breakdown) in mice brainstems from ventral and coronal positions are shown (5x and 2.5x objective, *n* = 5 pr. group). (**B**) Bar plots showing activated and suppressed terms associated with cellular adhesion and junctions, derived from GSEA analysis of conserved DEGs in GeoMx spatial profiles and single-cell RNA sequencing profiles. (**C**,** D**) Comparison of enriched function term of up- and downregulated genes in endothelial cell. (**E**) Plots of receptor-ligand communication pairs with notably differential expression specifically on day 6 post-infection, involving endothelial cells as receivers during the course of HSV-1 infection: the red and blue dot plots represent per-sample pseudobulk expression of communication pairs. (**F**) Heatmap showing the prevalence of each endothelial cell subpopulations, estimated by the Ro/e score. The numbers on the left indicate the subpopulation’s rank, with lower numbers indicating higher abundance and higher numbers indicating lower abundance. (**G**) Heatmap presenting the characteristic marker genes expression pattern in endothelial cell subpopulations. (**H-I**) Dot plot showing the expression of pyroptosis and apoptosis marker genes in endothelial cell subpopulations. (**J**) Violin plot of selected receiver genes expression in subpopulations of endothelial cells. (**K**) Violin plot of selected ligand genes expression in subpopulations of microglia and monocytes

The early analysis of the scRNA transcriptomes revealed a significant reduction in cells associated with the BBB, i.e. astrocytes, pericytes, and endothelial cells (Fig. S1H). Upon examining for access of Evans blue into the brain stem in HSV-1-infected mice, we observed that the exclusion of the dye in uninfected mice was impaired following infection, notable visible on day 6 and 8 post infection (Fig. 6A). Additionally, since the scRNA and GeoMx transcriptome data from the region around the brainstem principal trigeminal nucleus showed very strong correlation (Fig. S6A), and enrichment of the BBB integrity GO terms (Fig. 6B), we reasoned that the scRNA sequencing data could be used to infer transcriptional changes and cell-cell communications in the BBB microenvironment. Focusing on endothelial cells, we found strong upregulation of IFN responses throughout the time span of the experiments, including both type I and II IFNs, and a later upregulation of processes supporting T cell responses (Fig. 6C, Fig. S6B). Importantly, however, at late times we observed downregulation of pathways associated with tissue homeostasis and integrity, as well as downregulation of pathways involved in interaction between the neurovascular unit and neurons (Fig. 6D).

In order to infer cell-cell communications responsible for the observed changes we used NicheNet. First, we noted that the crosstalk between cells forming the BBB was impaired in the infected brain stem (Fig. 6E, Fig. S6C). This included for instance the interactions between astrocytes and endothelial cells through Vascular Endothelial Growth Factor A (VEGFA) and its receptors as well as between pericytes and endothelial cells through Transforming growth factor beta (TGFβ) and angiopoietins (Fig. S6C). Second, we found that the inflammation evoked by the infection engaged endothelial cells in several cell-cell interactions known to promote break-down of the BBB. Most notably *Tnfa*, mainly expressed by microglia and monocytes, but also other cell types (Fig. 2E), extensively stimulated endothelial cells (Fig. 6E, Fig. S6C-D). Additionally, *Ifng* expression by T cells was accompanied by induction of IFN-γ-induced gene profiles in endothelial cells (Fig. 6E, Fig. S6C). These results identify pathways that likely contribute to disturbance of the BBB during HSV-1 CNS infection.

Next, we subdivided the endothelial cells into subpopulations and identified 24 transcriptionally distinct clusters (Fig. S6E). In line with the observed break-down of BBB integrity, the major changes in subpopulation composition occurred on day 6 and 8 post infection, with emergence of populations #8, #22, and #24, and to a lesser extent population #14 and #18 (Fig. 6F, Table S3). Several of these populations were categorized as disease- or damage-associated phenotypes (Fig. S6F), although they generally did not contain high levels of viral transcripts (Fig. S6G). This suggests that the damage to the BBB is caused by inflammation rather than viral cytopathic effects. In line with the disease- or damage-associated phenotypes and involvement in disturbance of BBB integrity, populations #18, #22, and #24 showed decreased expression of tight-junction and high expression of several genes involved in pyroptosis and apoptosis (Fig. 6G-I). This was also seen in astrocytes, which were not examined in detail, but expressed high levels of *Gsdme*, encoding a death executer in pyroptosis (Fig. S6H). Endothelial cell subpopulation #14 exhibited a proliferative transcriptome profile, potentially indicative of initiation of repair activities. Finally, when looking into the cell-cell crosstalk between subpopulations in the different cell types, we found that IFN-γ and TNFα receptors *Ifngr2* and *Tnfrsf1a* were highly expressed in the endothelial subpopulations suspected of being involved in BBB damage, including #18 and #22 (Fig. 6J). These cells received cytokine signals from microglia subpopulation #14 and multiple monocyte subpopulations, including #7, #13, and #14 (Fig. 6K).

Collectively, these data show that HSV-1 infection in the CNS evokes an inflammatory response, which disturbs the steady-state activity of the brain vasculature and breakdown of the BBB, thus facilitating influx of immune cells in the brain, but also allowing influx of unwanted molecules and alterations of homeostatic interactions in the neurovascular unit.

### Disturbance of neuro-physiological brain activities

**Fig. 7:**
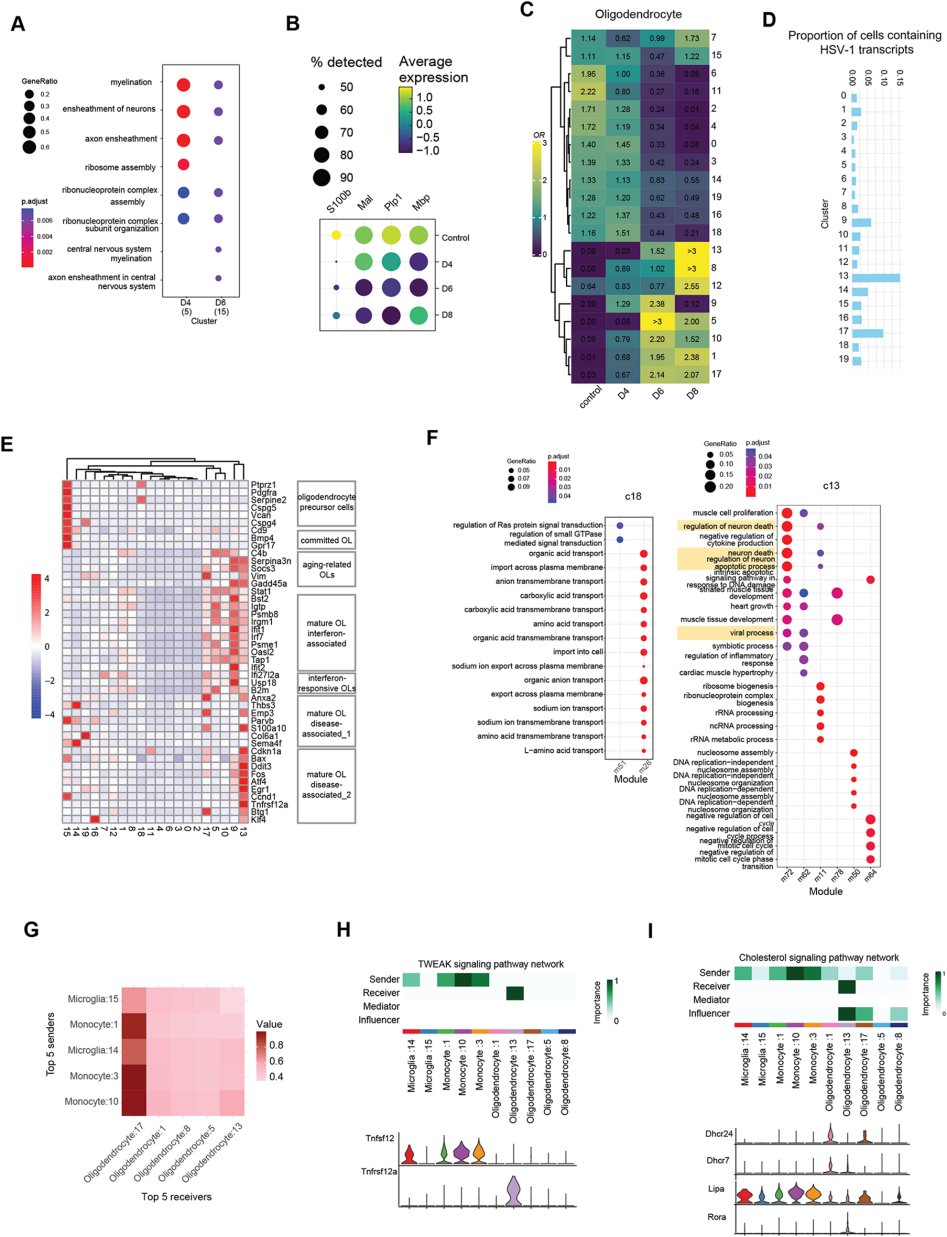
Oligodendrocyte dysfunction and differentiation impairment induced by HSV-1 infection. (**A**) Dot plot illustrating the enriched functional terms of downregulated genes of oligodendrocyte following infection. The size of each dot represents the gene ratio associated with each enriched term, while the color intensity indicates the statistical significance of the enrichment. (**B**) Dot plot showing the expression patterns of selected genes. The size of each dot represents the proportion of cells expressing the gene, while the color intensity reflects the gene expression level. (**C**) Heatmap showing the prevalence of oligodendrocyte subpopulations at different time points, estimated by the Ro/e score. The numbers on the left indicate the subpopulation’s rank, with lower numbers indicating higher abundance and higher numbers indicating lower abundance. (**D**) Bar plots showing the proportion of cells containing viral transcripts. (**E**) Heatmap of reported marker genes for oligodendrocyte subpopulations associated with specific labeled phenotypes. (**F**) Dot plots illustrating enriched GO terms in oligodendrocyte subpopulations #18 and #13. (**G**) Heatmap displaying the top 5 communication subpopulations with oligodendrocytes as receivers on day 8 and microglia, and monocyte subpopulations as senders. (**H**,** I**) Heatmap displaying the network centrality scores of TWEAK and Cholesterol signaling pathway. Violin plots illustrating gene expression distribution of TWEAK and Cholesterol signaling

A second observation from the global transcriptome analysis was disruption of physiological brain activities at late time points post infection, even after virus had been eliminated (Fig. 1E). To explore the underlying basis for this, we interrogated in which cell types the associated transcriptomic alterations occurred. Interestingly, we found transcriptional changes in oligodendrocytes to mainly contribute to the enrichment of terms associated with disturbance on physiological brain activities (Fig. 7A, Fig. S7A). Our scRNA dataset did not include many neurons, which are frequently lost during the isolation of single cells [45]. However, interrogation of the GeoMx-based transcriptome of the HSV-1-infected areas of the brain stem, which abundantly contained neurons (Fig. 5A), provided support for the alterations in neuro-physiological pathways (Fig. S7B). Focusing more on oligodendrocytes, we found that the expression of key myelin-associated genes was significantly decreased during and after HSV-1 infection of the brain stem (Fig. 7B, Fig. S7C, D). Subcluster analysis revealed 20 distinct oligodendrocytes subpopulations (Fig. 7C, Table S3). Subpopulation #13 had the highest content of viral transcripts among the oligodendrocytes (Fig. 7D), with the transcripts being detected in the subpopulation on day 6 and barely on day 8. Subpopulation #15 was identified as a potential oligodendrocyte progenitor, subpopulation #9 as the most strongly IFN-responsive, and subpopulation #13 as disease-associated (Fig. 7E). A comparison of enriched functions among the subpopulations reveals demyelination following infection, with evidence of gliogenesis-driven recovery by subpopulations #8 and #5 (Fig. S7E).

Next, we reconstructed trajectories of oligodendrocytes following HSV-1 infection using variable gene expression and RNA velocity analysis (Fig. S7F-H). Gene modules uniquely expressed in each sub-partition of the trajectory revealed the transition from subpopulation #4 to #9, accompanied by a functional shift towards viral response and type I IFN signaling (Fig. S7I-K). Importantly, the transition from subpopulation #18 to #13 revealed a functional shift towards viral processes including genes promoting and repressing viral replication and control of cell death pathways, including and neuronal apoptosis (Fig. 7F, Fig. S7I, L). Based on predicted key transcription factors, *Cebpd*, *Stat3*, *Ddit3*, and *Pou3f1* were upregulated in subpopulations #9 and #13 (Fig. S7M). These factors are involved in inflammatory signaling and stress-induced apoptosis. Moreover, we noted that the transition from subpopulation #18 to #13 was associated with reduced expression of Pou3f2, which is associated with neurodevelopment.

Finally, we explored the cell-cell communication network engaged by oligodendrocytes. Five subpopulations were identified as the top signaling receivers day 8 after infection, including #13, and were inferred to receive their signals from monocyte and microglia subpopulations (Fig. 7G). The top receiving oligodendrocyte cell population was #17, which was the second most virus RNA-rich population, of mid-high abundance on day 6 and 8 post infection, and associated with both IFN- and disease-related phenotypes (Fig. 7C-E). Analysis of the incoming signaling patterns in oligodendrocytes revealed that signaling by the TNF superfamily member TWEAK/TNFSF12 and cholesterol metabolism pathway were specifically upregulated in oligodendrocyte subpopulation #13 (Fig. 7H-I, Fig. S7N). The signals were received from the top sensing monocyte and microglial subpopulations (Fig. 7H-I). Collectively, the infection and inflammatory microenvironment, drives development of oligodendrocyte subpopulations with altered neuron myelination functionality and potentially promotion of neuronal cell death.

### Comparison of HSV-1 infection to other acute and chronic brain conditions

**Fig. 8:**
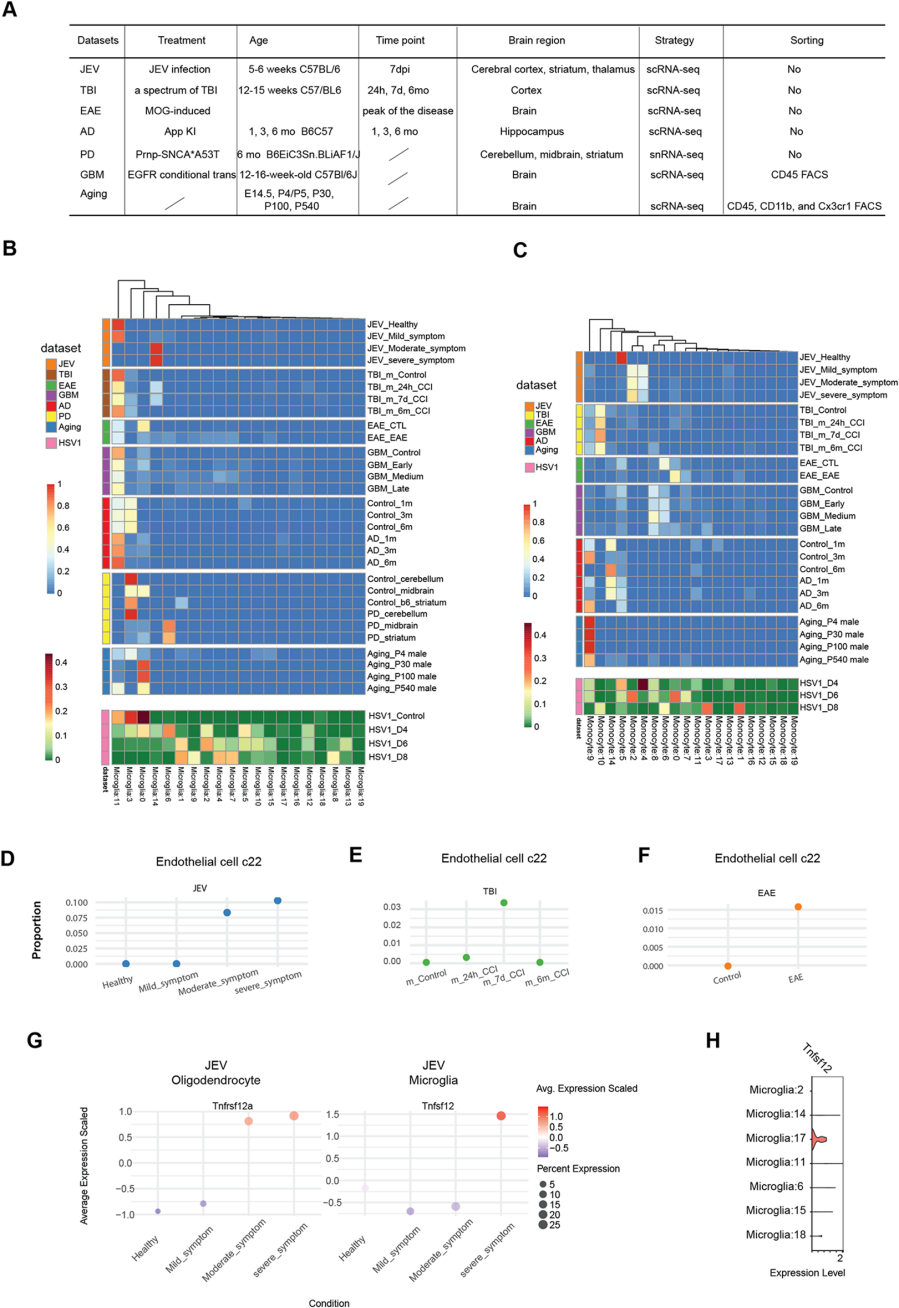
Presentation of virus-responsive subpopulations in diverse brain pathology datasets. (**A**) Summary of collected published datasets, including treatment designs. (**B**,** C**) Heatmaps showing the proportions of microglia and monocyte subpopulations found in our study compared across different conditions in the collected published datasets. The subpopulation number corresponds to the ones used in Figs. 3 and 4. (**D-F**) Proportion changes of endothelial cell subpopulation #22 in JEV, TBI and EAE datasets. (**G**) TWEAK signaling pathway genes expression pattern in oligodendrocyte and microglia from JEV dataset. (**H**) *Tnfsf12* gene expression pattern in microglia subpopulations from JEV dataset

As a final part of the study, we wanted to compare the identified brain immune population landscape from our study with datasets reported from other studies in a broad panel of acute and chronic brain diseases (Fig. 8A) [16, 46–51]. The datasets were mapped to HSV-1 single-cell transcriptomic profiles to compare the cellular composition and abundance of each subpopulation (Fig. S8A). Strikingly for microglia, we noted that similar to the early stages of HSV-1, subpopulation #14 was highly abundant in the JEV-infected mouse brain correlating with disease severity (Fig. 8B, Fig. S8B). As described above, this population showed high levels of viral transcripts, activity of IFN, cytokine, and chemokine pathways (Fig. 3I-J, 7F-G). Microglia subpopulation #14 was also detected, at lower abundance in the acute stages after traumatic brain injury (TBI) but was not the most abundant microglia population (Fig. 8B, Fig. S8C). For other brain diseases, we observed relatively little overlap with the microglia profiles found in the HSV-1 mouse model. One noticeable exception was subpopulation #6, which was also detected in midbrain and striatum of a Parkinson’s disease transgenic mouse model [50]. This population was active for cytokine signaling, but not chemokine activity (Fig. 3J, 7F-G). These data therefore suggest that microglia subpopulation #14 is particularly enriched in acute viral infections and may therefore tentatively be called virus-activated microglia (VAM).

For the monocytes, we saw a more diverse pattern of distribution of subpopulations between diseases (Fig. 8C). Despite this, the JEV infection showed a high abundance of subpopulations #2 and #4, similar to the HSV-1 infection (Fig. 8C, Fig. S8B). However, other subpopulations readily detected in the HSV-1-infected brain stem (Fig. 4A) were not found during JEV infection. While #4 is a characterized by expression of type I IFN genes and *Tnfa* (Fig. 4D), #2 has received signaling from IFN-γ and promotes antigen presentation (Fig. S4D). For other monocyte subpopulations, showing high abundance at least at one time point during HSV-1 infection, population #10 was interesting for its abundance in TBI (Fig. 8C, Fig. S8C). This population appears late during HSV-1 infection and is enriched for both inflammatory cytokine pathways, suppression of key inflammatory activities and tissue repair (Fig. S4G). Thus, the monocyte landscape during HSV-1 infection shows resemblance to that seen after JEV infection, and with some overlap with populations accumulating during acute sterile brain damage.

**Fig. 9:**
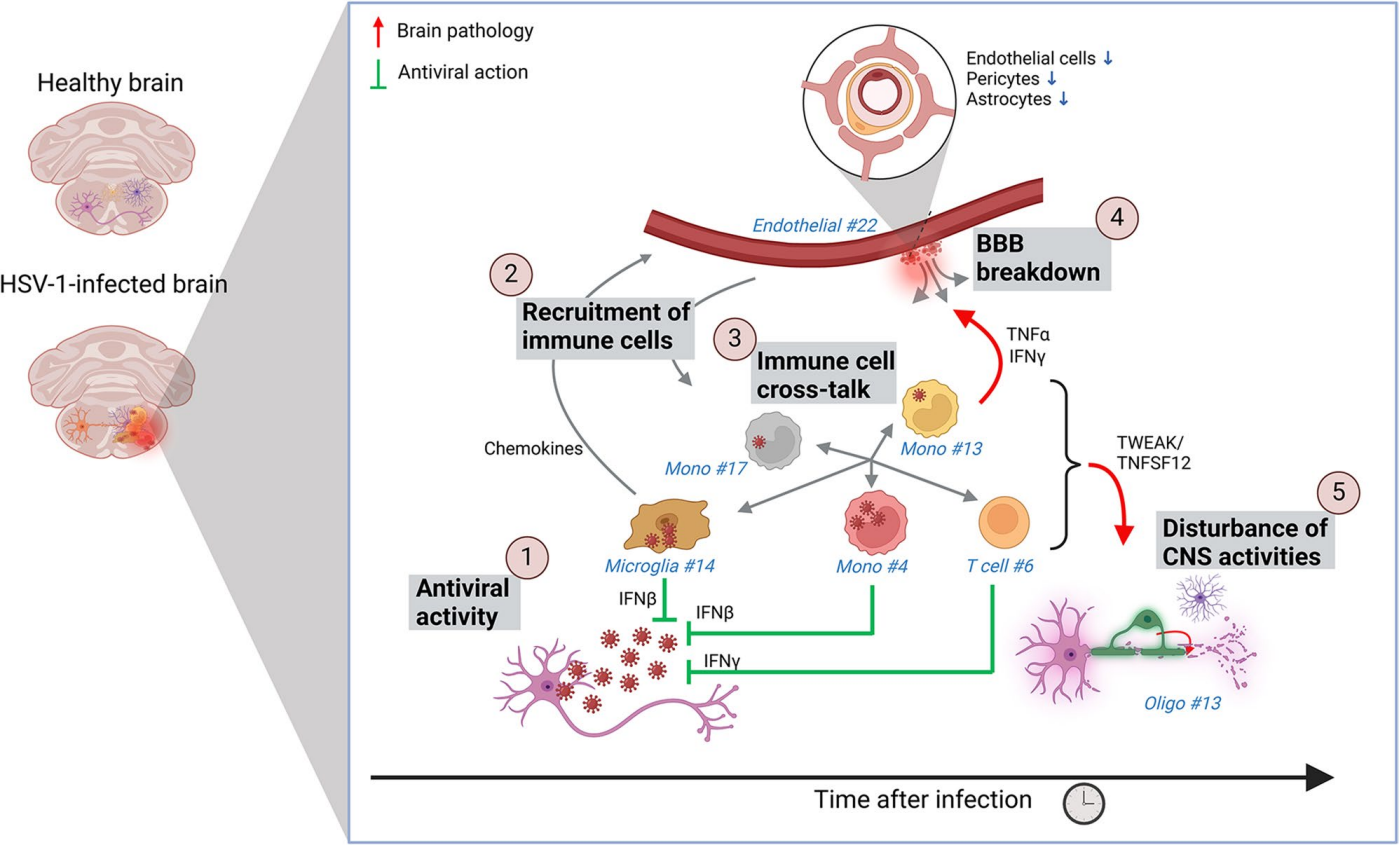
Model for the antiviral and inflammatory activities in the HSV-1-infected mouse brain. HSV-1 infection induces early expression of an innate antiviral IFN response, mainly driven by a microglia subpopulation, that is characteristic for virus infections (virus-activated microglia, VAM). This is paralleled by expression of cytokines and chemokines, which trigger recruitment of immune cells, mainly monocytes. The monocytes include an array of subpopulations, and engage in extensive cross-talk with other immune cell populations, thus amplifying their activities. Through the accompanying inflammatory response, microglia, monocytes, and T cells cross-talks with brain structures and functions, including the BBB and the oligodendrocyte-neuron unit. These interactions involve numerous pathways, including TNFα, IFNγ, and TNFSF12/TWEAK and lead to loss of BBB integrity and neuro-physiological functions. Eventually, this can cause acute disease, or long-term sequelae

When analyzing the datasets from the brain diseases for the observed disruption of the BBB, we focused on endothelial cell population #22, which showed high responsiveness to TNFα and IFN-γ signaling, and decrease of tight junction-related genes, and increase of death-related genes (Fig. 6G–J). Interestingly, this population was enriched in JEV infection correlating with disease severity (Fig. 8D) and was also enriched in the late acute stage of TBI and experimental autoimmune encephalomyelitis (EAE) (Fig. 8E, F). Finally, we wanted to explore whether the leukocyte-oligodendrocyte crosstalk through the TWEAK/TNFSF12 pathway was exclusive to the HSV-1 model or also observed in other disease models. We found that microglia-oligodendrocyte communication through the TWEAK/TNFSF12 pathway was also strongly induced in the JEV model correlating with disease severity (Fig. 8G), with microglia subpopulation #17 being a prime sender of signals (Fig. 8H). Beyond viral encephalitis, we found induction of the pathway in EAE (Fig. S8D), whereas for other brain diseases only limited parallel upregulation of microglial *Tnfsf12* and oligodendrocyte *Tnfrsf12a* was observed (Fig. S8E-G). Collectively, this analysis suggests that our findings with HSV-1 reveal a general pattern for viral brain infections with respect to both microglia and immune cell profiles and crosstalk with the brain tissue (See Fig. 9).

## Discussion

Viral brain infections can develop into devastating encephalitis, and can have fatal outcome, or lead to severe morbidity [1]. Moreover, viral brain infections can prime for post-infectious complications, including cognitive defects, headache etc [1]. The immune system is essential for controlling viral CNS infections but can also contribute to disease development [52, 53]. In this work, we show that HSV-1 infection in the CNS leads to a profound transcriptional change in many cell types, recruitment of a panel of immune cells, which drive both protective and pathological responses. In particular, we identify specific microglia and monocyte subpopulations, and uncover their phenotypic profiles and interaction networks, and suggest how they contribute to the outcome of infection. This includes both control of infection and disturbance of neuro-physiological functions, most notably the integrity of the BBB and proper functions along the glial-neuronal axis (Fig. 9).

An initial overall characterization of the transcriptional changes during the course of HSV-1 brain infection showed strong upregulation of immune- related genes, and most DEGs were indeed expressed by CD45 + cells, i.e. immune cells. The dominant cell types were, in addition to microglia, monocytes, and later T cells, all of which continued to increase after the infection was cleared, in agreement with previous reports [54]. At the single-gene level we observed the strongest correlation between viral transcripts and levels of *Il1rn* in single cells. Interestingly, this is in agreement with clinical data showing correlation between *IL1RA* and HSE disease [55], thus providing support for the translational potential of our results. We also found the transcriptome of the infected brain to contain elevated levels of antisense transcripts, and mismatch with the genome sequence. This is indicative of a stress-related transcriptional response in infected areas [56, 57]. Finally, we observed downregulation of a series of pathways involved in homeostatic brain activity. The latter is consistent with the clinical observation that the patients recovering from encephalitis may suffer several long-term effects, such as cognitive defects [58].

Microglia is the main brain-resident immune cell type and is well-described to express most pattern recognition receptors and proinflammatory cytokine receptors [59]. Previous studies have shown that microglia are essential for host defense against viral CNS infections with both RNA and DNA viruses [18, 60, 61]. Depletion of microglia was found to decreased expression of IFNβ and impaired activation of T cells [18, 60, 61]. In our study we observed increased abundance of microglia in the brain stem following HSV-1 infection, and also that microglia are a prime source of transcripts for *Ifnb1* and *Tnfa*, and to a lesser extent also *Il1b* and *Il6*. Interestingly, we observed that microglia undergo a profound and diverse change in subpopulation composition upon infection, with none of the subpopulations being present in the uninfected brain stem areas sequenced being detectable on day 6 post infection. Generally, we observed a dynamic change from homeostatic subpopulations to early interferonic/inflammatory subpopulations in the presence of virus, and eventually to proliferative and antigen-presenting subpopulations at late time points. Notably, microglia subpopulation #14 was highly abundant on day 4 p.i., expressed the highest levels of type I IFN and chemokine genes as well as *Tnfa*. This population showed the highest abundance for viral RNA among the microglia subpopulations, suggesting this subpopulation to be the key virus-sensing cell population, orchestrating IFN-mediated antiviral defense and recruitment of cells from the periphery. Microglia #14 was involved in numerous interactions, including endothelial cells and oligodendrocytes, thus indicating involvement in both activation of infiltrating immune cells, disruption of the BBB and proper neuronal support by oligodendrocyte. Given the importance of microglia in antiviral defense [18, 60, 61], it was interesting to note that subpopulation #14 was also highly enriched in JEV infection, and only to a lesser extent in TBI. The population was not abundantly present in other brain diseases examined, including chronic immunological diseases, glioblastoma, or neurodegenerative diseases. This suggests that microglia subpopulation #14 does in fact represent the virus-activated microglia, VAM, parallel to other defined microglia subpopulations such as the inflammation-associated microglia emerging following lipopolysaccharide exposure [62], and the disease-associated microglia found in neurodegenerative diseases [63].

Monocytes are not present in the brain during steady state, but are recruited in high numbers to the brain in response to infections with HSV-1 and other viruses [54, 64, 65], as well as non-viral acute brain condition, like trauma [66]. We found that the recruited monocytes underwent a highly dynamic change, illustrated by rapid change in the composition of subpopulations. At the early time-points, the monocytes were characterized by antiviral activity, but also alteration of the endothelium and glial cell activity. At later time points this shifted to crosstalk with T cells, including responsiveness to IFN-γ and antigen-presentation, as well as proliferation and regulatory processes. Regarding cell-cell crosstalk, we found that subpopulations #13, #17, and #7, among others, interacted with endothelial cells through multiple pathways, including TNF, IL-6, and IL1β and may thus contribute to the damage to the BBB. Moreover, we found populations that interacted extensively with oligodendrocytes to promote loss of functional support for neurons. This included the TWEAK/TNFSF12 pathway, previously reported to promote neuronal cell death [67]. Similar to microglia, the monocyte population that contained the most viral RNA, #4, also produced the highest levels of type I IFN genes. This population was also abundant in JEV infection. However, unlike microglia, expression of inflammatory cytokines did not correlate closely with levels of viral transcripts and was high in many subpopulations. This could indicate that the inflammatory response in monocytes is driven by more complex mechanisms than merely viral sensing by PRRs, possibly involving cytokine stimulation, metabolic alterations, and epigenetic alterations. In agreement with this, we observed monocytes to represent a more prolonged source of inflammatory cytokines and cell-cell communication beyond the duration of infection and may therefore contribute to post-infectious brain damage. This included for instance subpopulation #2, which was also highly abundant in JEV infection and exhibited a phenotype with cytokine signaling and positive regulation of T cell responses. The expression of inflammatory cytokines by monocytes not containing high amounts of viral RNA could also allow for monocyte-driven inflammatory activities in brain areas not directly infected by HSV-1. Of note, recent data based on HSV-1 infection in human brain organoids have identified TNFα as a key contributor damage of neuronal processes and neuroepithelium [35]. The role of recruited monocytes in antiviral defense and brain pathogenesis needs further investigation. Our work did not reveal trajectories for immune cell populations from acute infection to chronic/progressive disease states. This will likely require more extended and temporally resolved datasets but has the potential to reveal important information on inflammatory mechanisms and cues promoting brain pathology. This is interesting given the proposed link between infections, including HSV-1, and Alzheimer’s disease [4].

Disruption of the BBB is a hallmark of many severe brain diseases, including viral encephalitis [68, 69]. Breakdown of the BBB is associated with numerous pathological processes, including access of large biomolecules from the blood stream into the CNS, disturbance of the mechanisms ensuring delivery of oxygen and nutrients into the brain, facilitation of influx of immune cells into the CNS, and increased permeability of blood vessels leading to vasogenic edema [68]. HSE is known to cause severe cerebral edema, and we have previously reported this to occur in the infected part of the mouse brain brainstem [12]. Here, we found that the brains of HSV-1-infected mice were permissive to Evans blue, and also that there was an unproportionally large loss of BBB-associated cell types in the infected brains. Previous studies have reported that brain infections with JEV, rabies virus, coronavirus, reovirus, and WNV also leads to breakdown of the BBB [36, 70–73]. For most of these infection models, IFN-γ contributed to the disruption of the BBB through downregulation of tight junctions [70–73], whereas in the WNV model, it was dependent on TNFα signaling [36]. Yet other studies have implicated a role for IL1β in BBB breakdown, including activation of the endothelium and recruitment of activated proinflammatory leukocytes [74, 75]. We observed high expression of a range of genes involved in programmed cell death, including pyroptosis and apoptosis, in the endothelial cell populations emerging late during infected and exhibiting disease/damage-associated transcriptome profiles. As we observed only low abundance of viral RNA in BBB-associated cells, we favor the explanation that the loss is mainly caused by inflammatory mechanisms, and not direct lytic virus replication. The disruption of BBB integrity in the HSV-1-infected brain may involve several mechanisms. First, rapid and prolonged downregulation of crosstalk that generates and maintains the BBB, including TGFβ, VEGFA and Angiopoietin signaling, from different cell types towards endothelial cells. Second, downregulation of a panel of tight-junction-related genes. Third, elevated inflammatory signaling to endothelial cells, notably IFN-γ signaling from T cells and TNFα, IL1β, and IL-6 signaling from specific monocytes and microglia subpopulations, may promote disruption of barrier integrity. Fourth, adhesion of activated proinflammatory leukocytes, which produce e.g. inflammatory cytokines, reactive oxygen species, or matrix metalloproteases. Fifth, loss of BBB-associated cell types, likely involving different forms of programmed cell death. The points listed above may well be mechanistically connected. Since the most dominant inferred cell-cell communications to endothelial cells were also found in a dataset from mice infected with JEV, we propose that the observed processes associated with disruption of the BBB may be general for acute viral brain infections.

Pathway enrichment of the single-cell and GeoMx spatial transcriptomes revealed that differentiation of glial cells, myelination, and key neuronal activities were down-modulated late during and early after infection. The downregulation of glia cell differentiation was mainly ascribed to activity of microglia, including subpopulation #14. Of note, this subpopulation was additionally involved in the crosstalk with endothelial cells at the BBB, but also the antiviral response. The microglia-dependent impairment of glial cell differentiation pathways is likely to delay brain repair after acute infection [76, 77]. Second, we found that the process of axon enchantment and myelination was down-modulated following infection. In particular, we identified one oligodendrocyte subpopulation #13, which underwent a trajectory towards promotion of neuronal death emerging late during infection and being highly enriched after clearance of the virus. This subpopulation received extensive signals from microglia and monocyte subpopulations through the TNF receptor super family member TWEAK/TNFSF12, which has previously been reported to mediate neuronal death in cerebral ischemia [67]. When examining scRNA sequencing datasets from other brain disease mouse models, we found similar immune cell-oligodendrocyte crosstalk in JEV infection and to a lesser extent EAE, but not other diseases. A number of viral infections can induce demyelination as a central feature of their neuropathology [78]. Future work should explore the physiological importance of the TWEAK/TNFSF12 pathway in disruption of myelination after acute brain infection. Collectively, these data underscore the dual nature of immune responses in the brain and highlight the importance of both imposing thresholds for activation of immune responses and for minimizing the duration of such responses.

In this work, we report a temporally resolved overview of the single-cell transcriptome during HSV-1 infection in the CNS. Through this analysis we identify a novel microglia subpopulation that is likely a central mediator of the early antiviral response, and recruitment of immune cells from the periphery. We also identify activities of microglia and monocytes subpopulations that contribute to brain pathology. This includes crosstalk with endothelial cells associated with disruption of BBB integrity and oligodendrocytes associated with neuronal myelination. In our study we used a model for non-lethal brain infections, thus allowing us to identify both beneficial defense activities as well as responses mediating transient or long-term brain pathology. In fact, several of the immune cell populations identified to exert the strongest communication with brain cells were present only transiently in this model. Our work may therefore highlight the importance of temporal restriction of the inflammatory response in the brain, including specific immune cell subtypes, to allow rapid re-establishment of homeostasis after infection. HSV-1 infection in the brain can range from milder infection with post infectious brain conditions to lethal HSE, and this work has now uncovered the single-cell and temporal overview of key pathways regulated during this infection. This knowledge will advance the understanding of how viral brain diseases can cause acute disease or prime for post-infectious brain conditions.

## Electronic supplementary material

Below is the link to the electronic supplementary material.

Fig. S1. Characterization of single-cell sequencing data from HSV-infected brain.

Fig. S2. Pathway enrichment in different cell types in the HSV-1-infected mouse brain.

Fig. S3. Analysis of microglia subpopulations during HSV-1 infection.

Fig. S4. Analysis of monocyte subpopulations in the HSV-1-infected mouse brain.

Fig. S5. Analysis of interactions of immune cells in the central immune system.

Fig. S6. Analysis of endothelial cells in the HSV-1-infected mouse brain.

Fig. S7. Analysis of oligodendrocyte subpopulations.

Fig. S8. Analysis of microglia and monocyte subpopulation representation in published datasets.

Table S1: GeoMx: Overview of ROI selections and raw count.

Table S2: scRNA: Quality control, viral reads and pct. of viral RNA-positive cells.

Table S3. Lists of differentially regulated genes, GO terms and DEGs at the cell type level.

Supplementary Figure legends

## Data Availability

All raw sequencing data and processed data have been uploaded to the NCBI’s Gene Expression Omnibus database (accession number: GSE283518). Any additional information required to reanalyze the data reported in this paper is available from the lead contact upon request.
