# Supplementary figures and images for: Temporally resolved single-cell RNA sequencing reveals protective and pathological responses during herpes simplex virus CNS infection
Source: J Neuroinflammation. 2025 May 31;22:146. doi: 10.1186/s12974-025-03471-x (PMC12125739; doi:10.1186/s12974-025-03471-x)

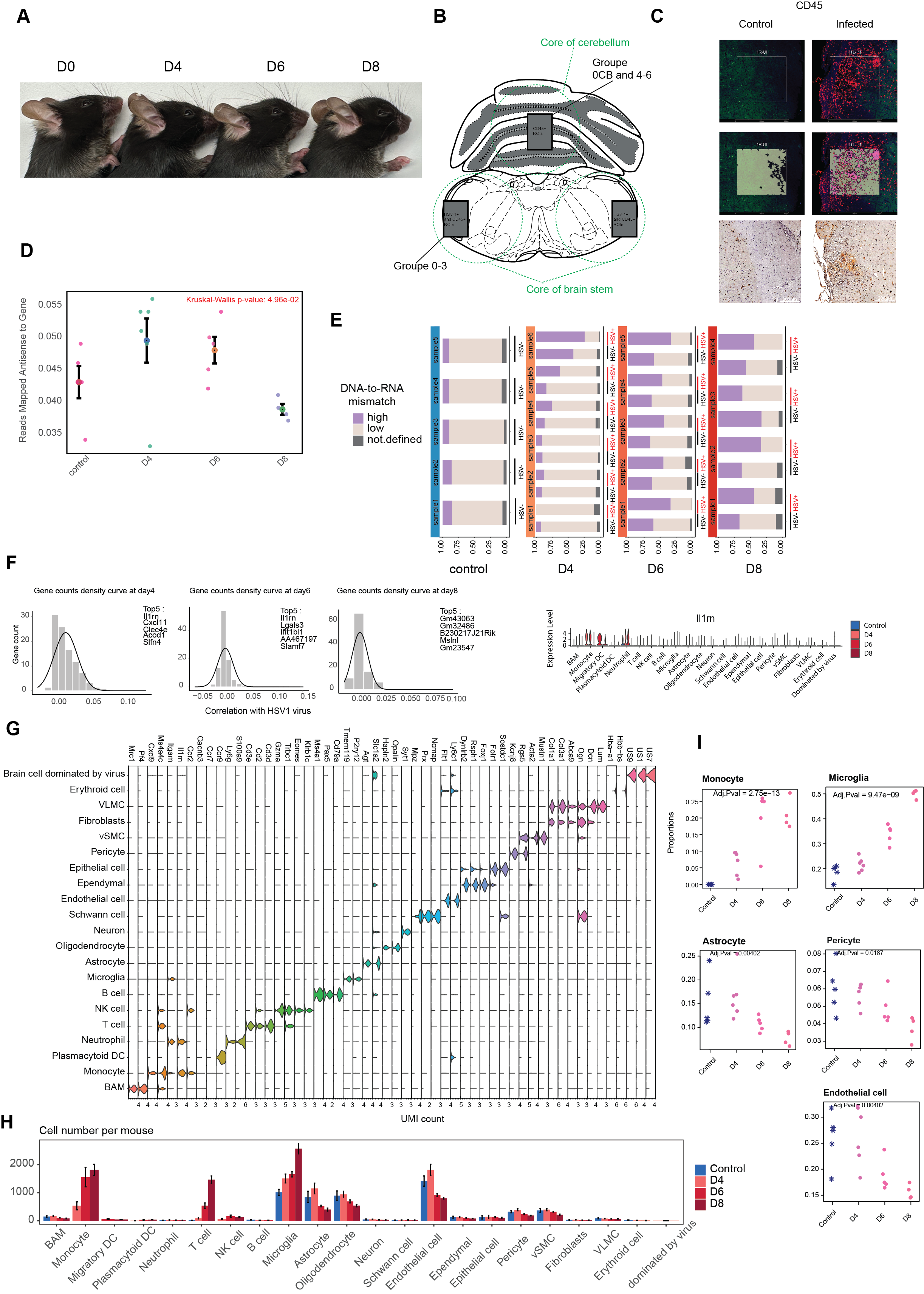

Supplement: Supplementary file 1 — Fig. S1. Characterization of single-cell sequencing data from HSV-infected brain. [file 12974_2025_3471_MOESM8_ESM.tif]

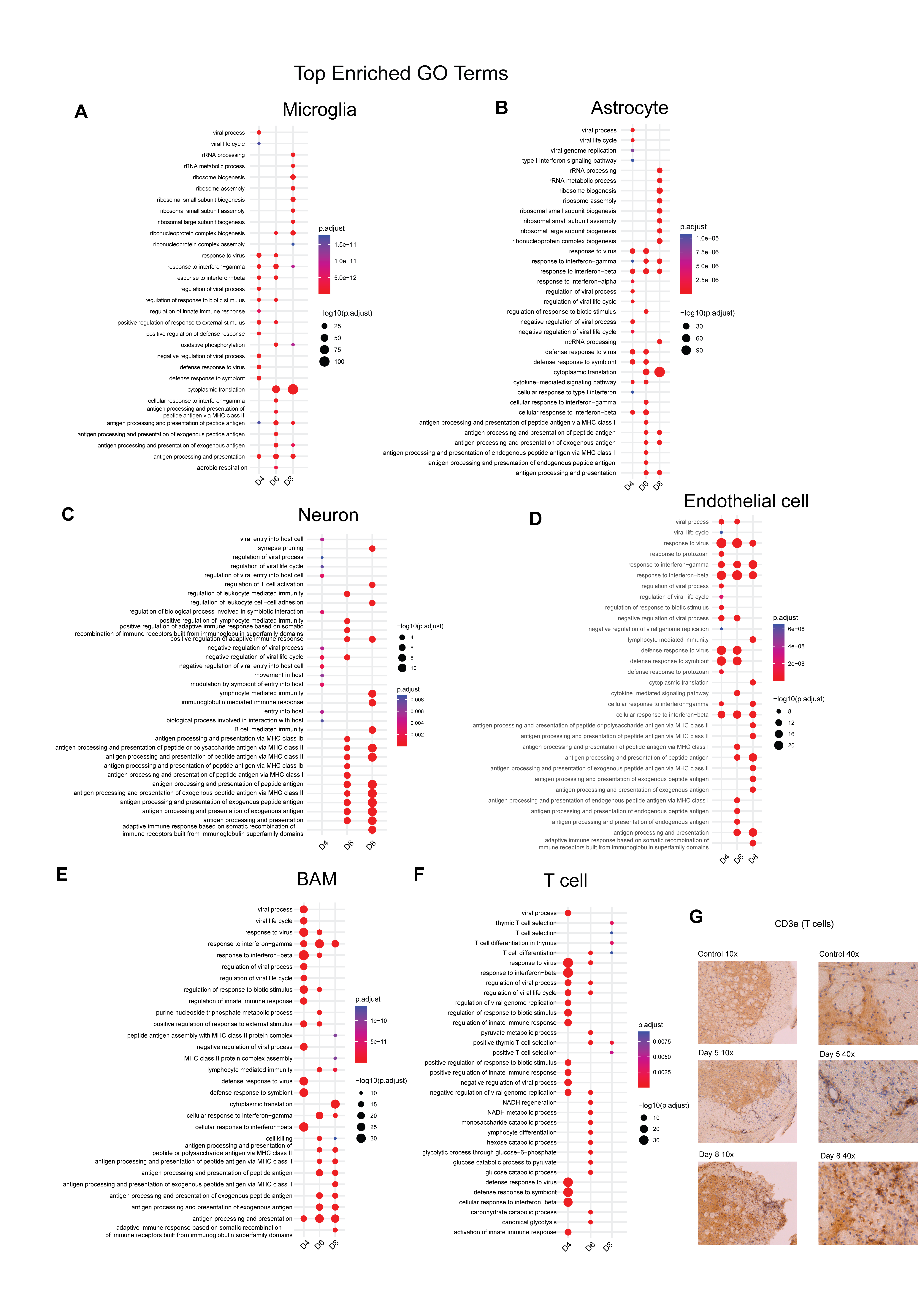

Supplement: Supplementary file 2 — Fig. S2. Pathway enrichment in different cell types in the HSV-1-infected mouse brain. [file 12974_2025_3471_MOESM1_ESM.tif]

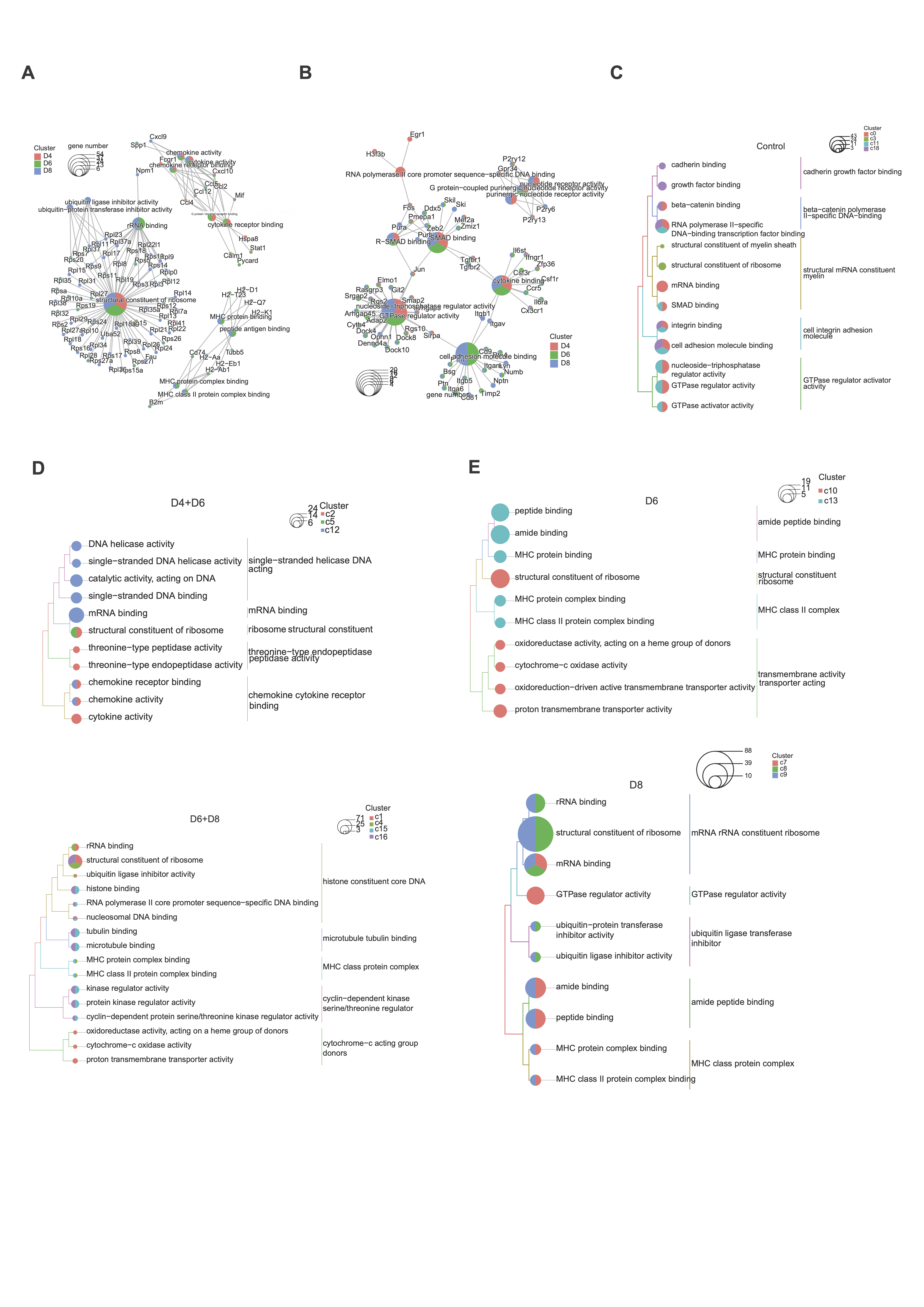

Supplement: Supplementary file 3 — Fig. S3. Analysis of microglia subpopulations during HSV-1 infection. [file 12974_2025_3471_MOESM2_ESM.tif]

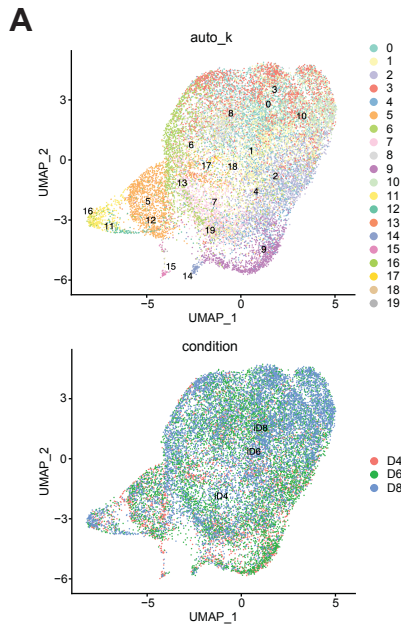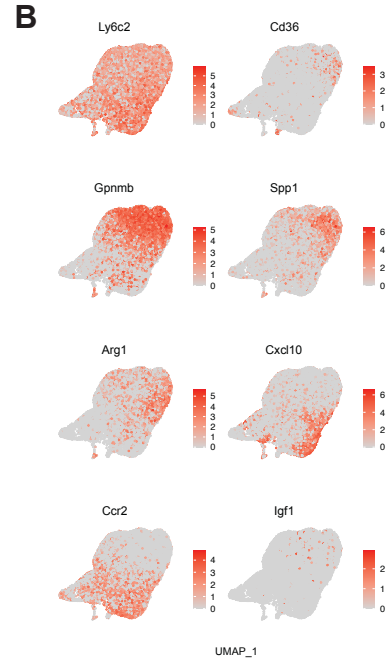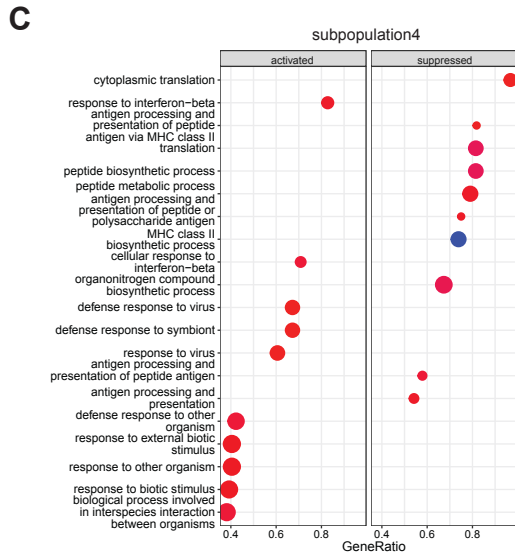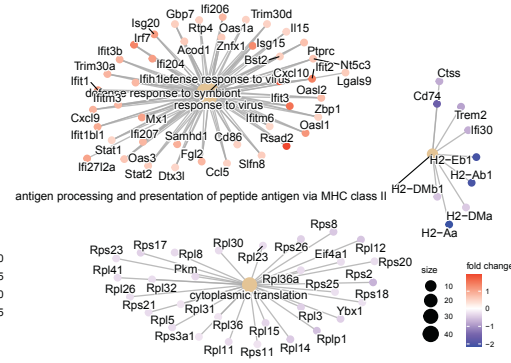

**D**

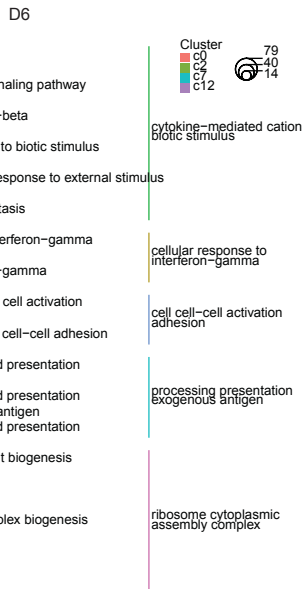

**E**

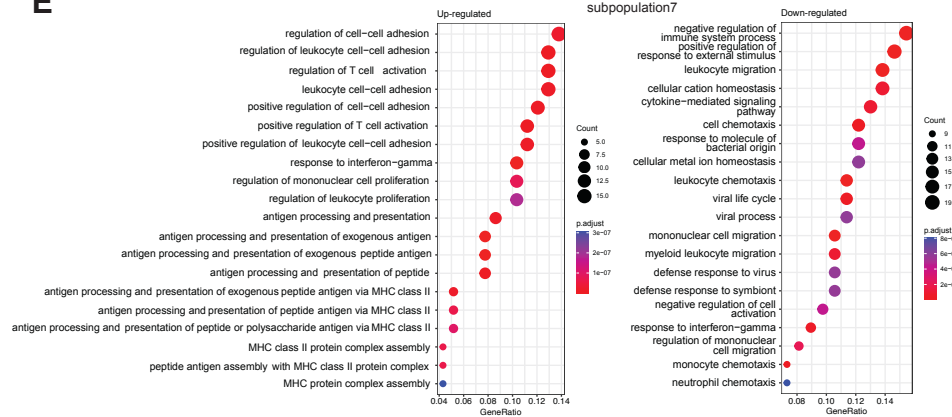

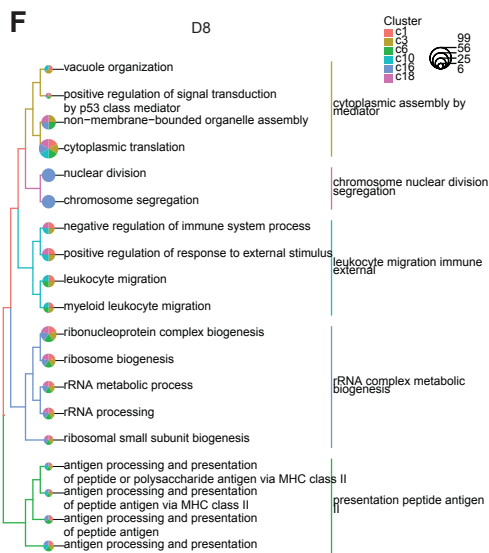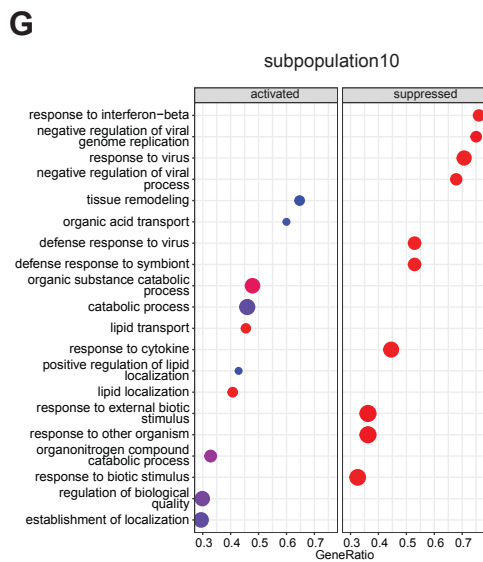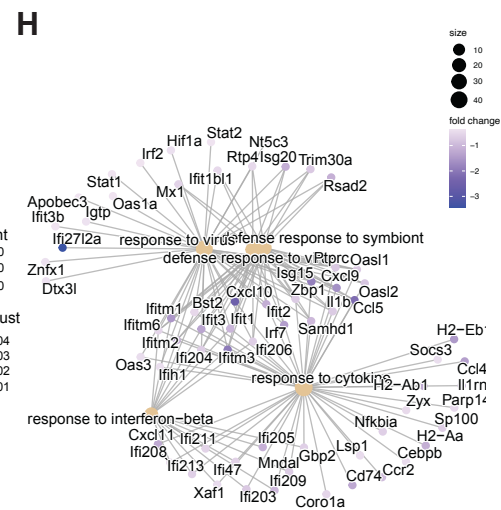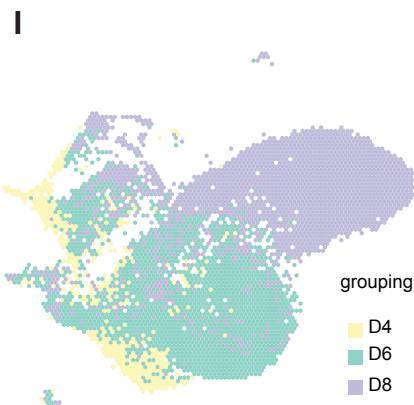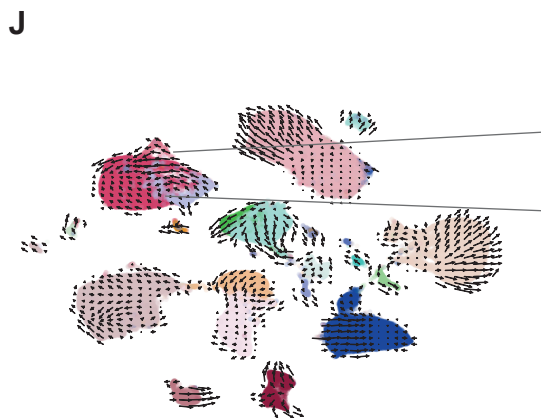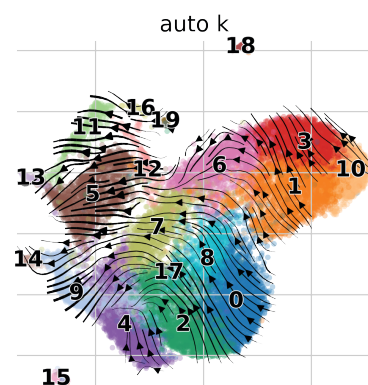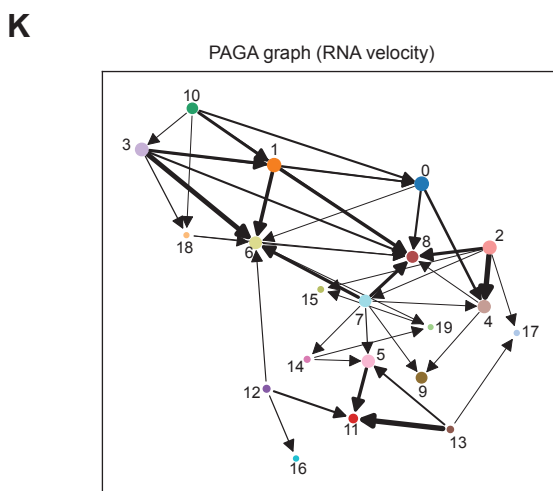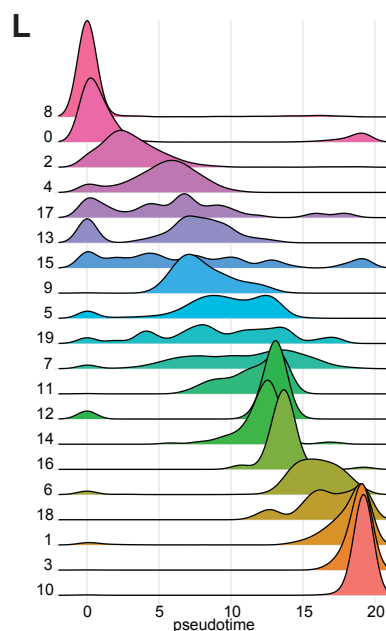

Supplement: Supplementary file 4 — Fig. S4. Analysis of monocyte subpopulations in the HSV-1-infected mouse brain. [file 12974_2025_3471_MOESM14_ESM.pdf]

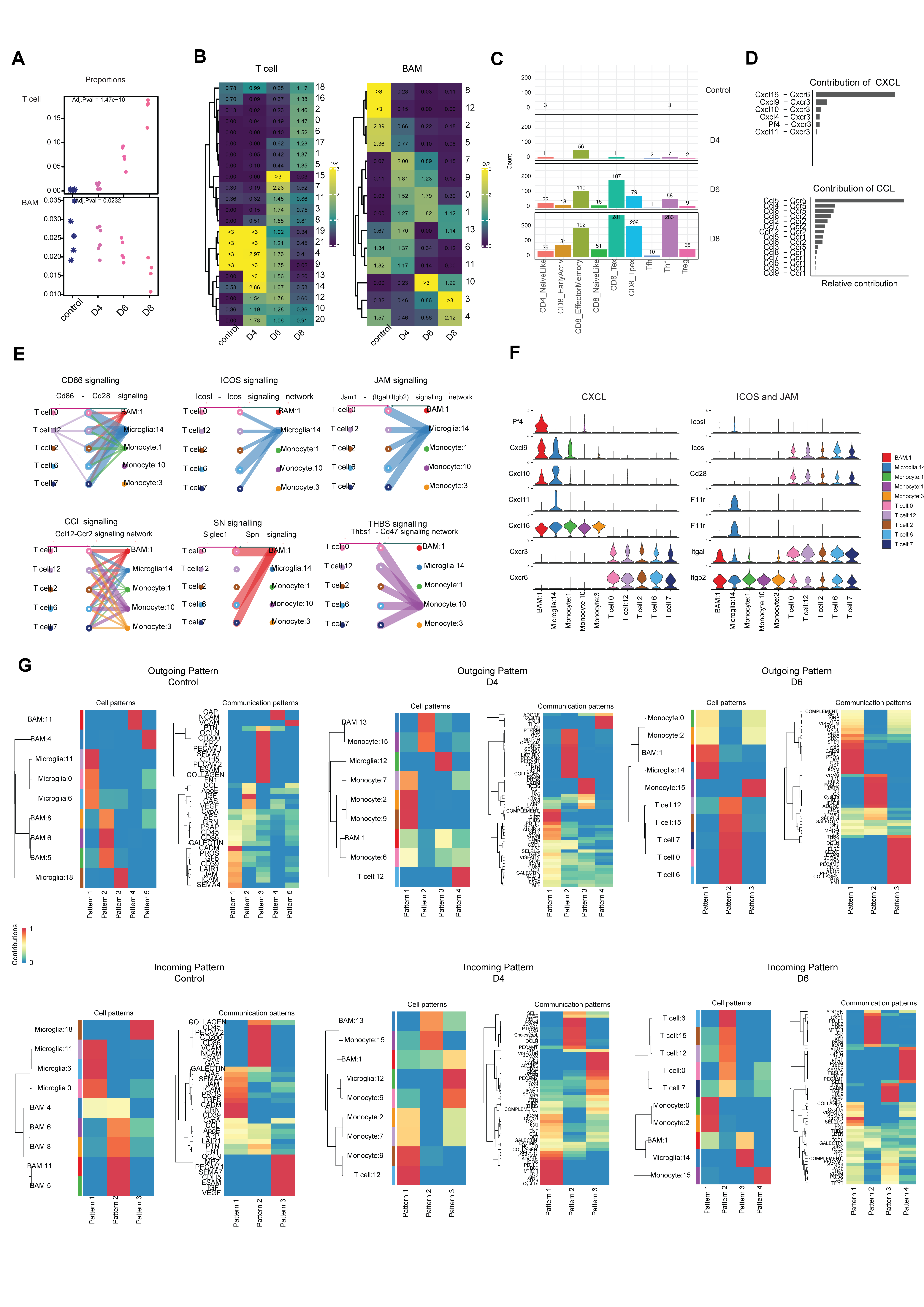

Supplement: Supplementary file 5 — Fig. S5. Analysis of interactions of immune cells in the central immune system. [file 12974_2025_3471_MOESM4_ESM.tif]

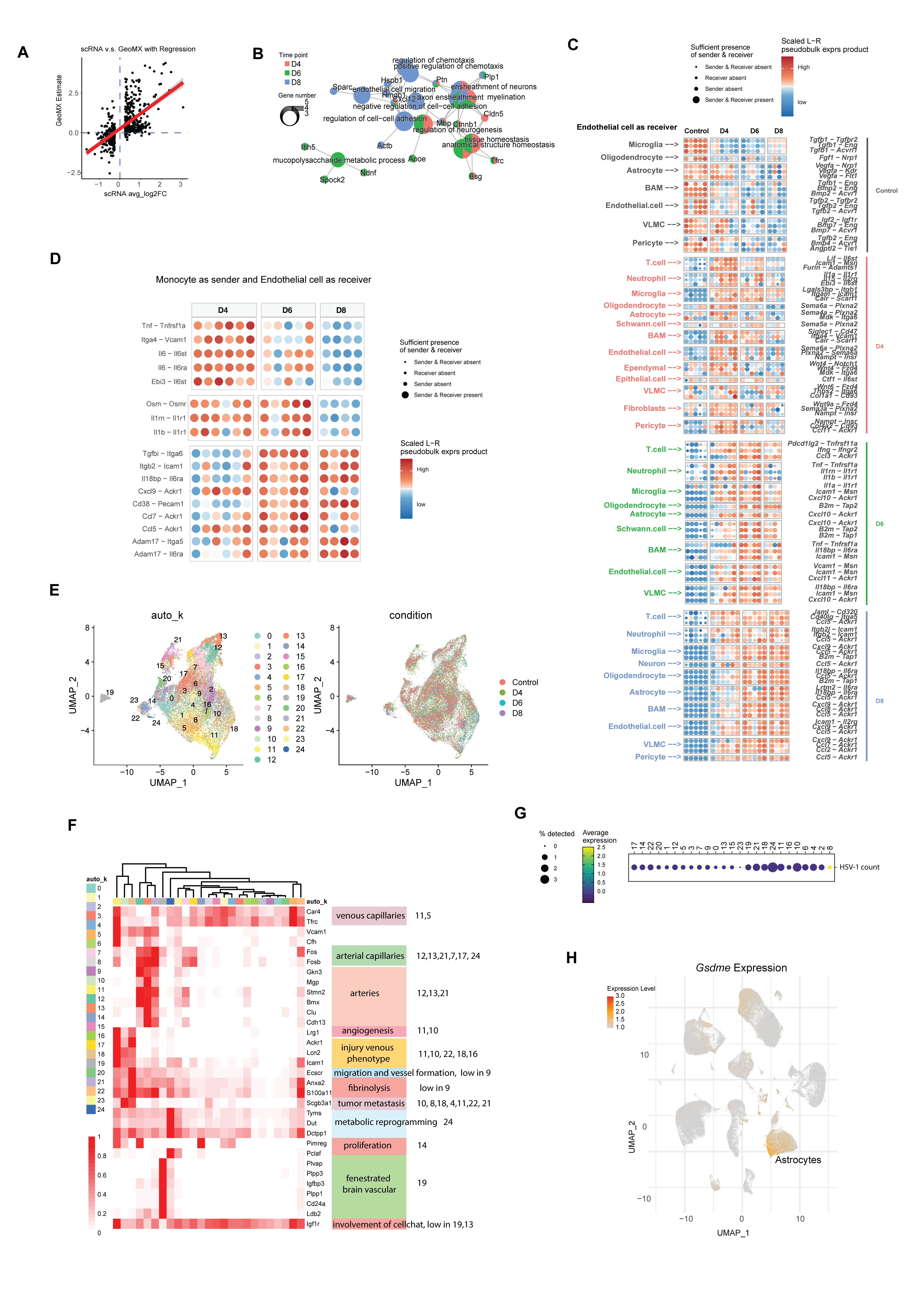

Supplement: Supplementary file 6 — Fig. S6. Analysis of endothelial cells in the HSV-1-infected mouse brain. [file 12974_2025_3471_MOESM5_ESM.tif]

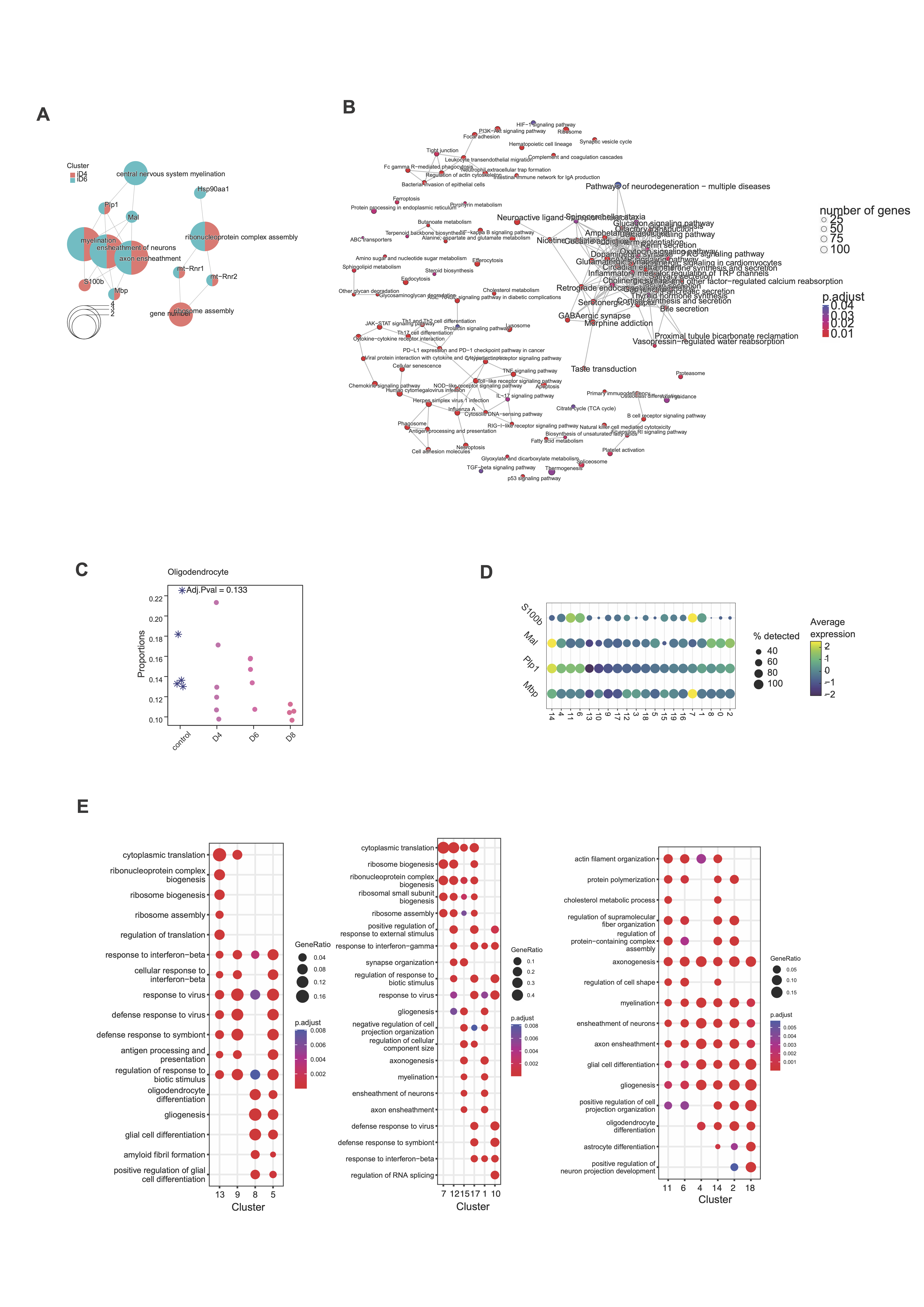

Supplement: Supplementary file 7 — Fig. S7. Analysis of oligodendrocyte subpopulations. [file 12974_2025_3471_MOESM6_ESM.tif]

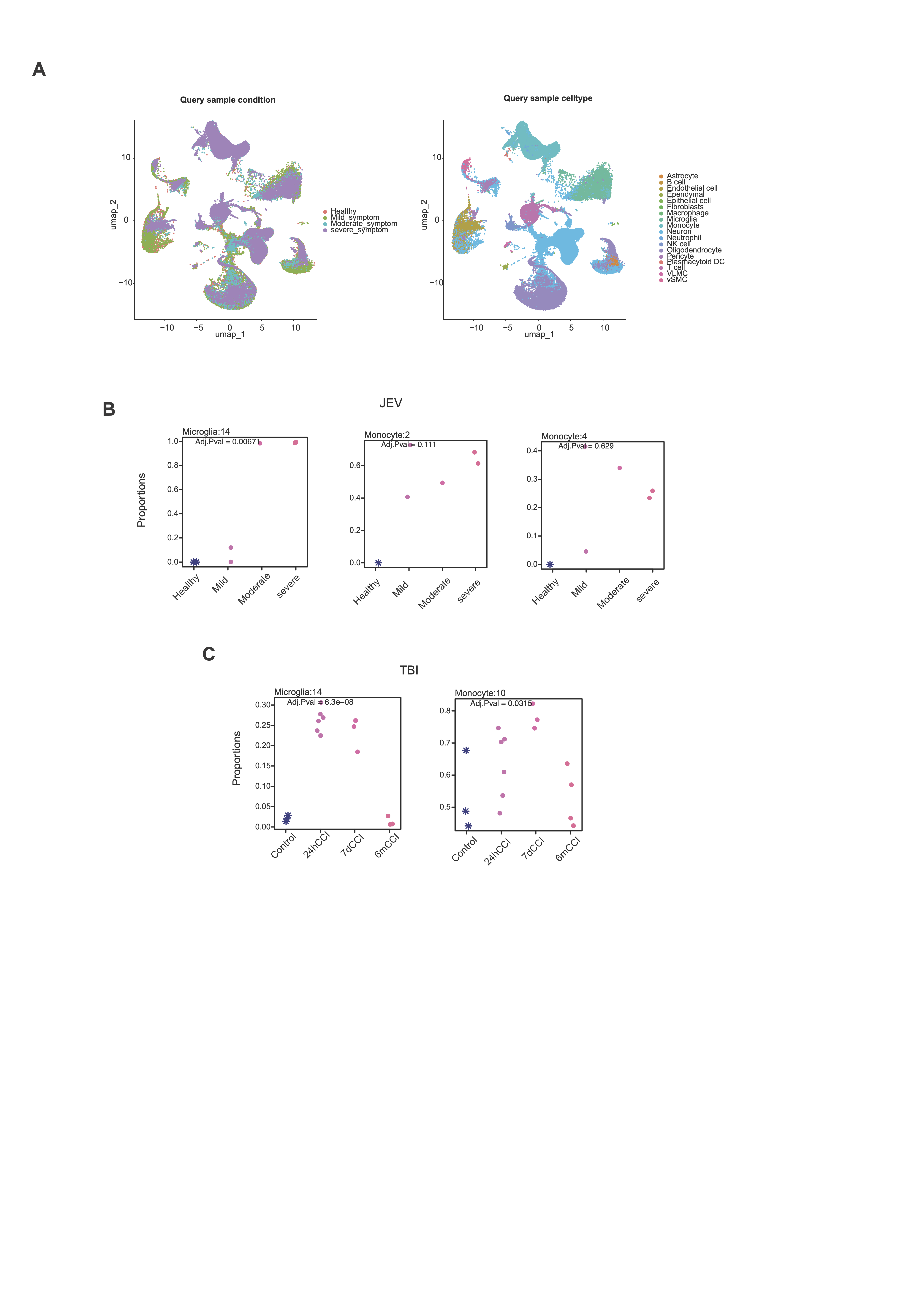

Supplement: Supplementary file 8 — Fig. S8. Analysis of microglia and monocyte subpopulation representation in published datasets. [file 12974_2025_3471_MOESM7_ESM.tif]
