# Supplementary material for: Temporally resolved single-cell RNA sequencing reveals protective and pathological responses during herpes simplex virus CNS infection
Source: J Neuroinflammation. 2025 May 31;22:146. doi: 10.1186/s12974-025-03471-x (PMC12125739; doi:10.1186/s12974-025-03471-x)
Supplement: Supplementary file 12 — Supplementary Figure legends [file 12974_2025_3471_MOESM13_ESM.pdf]

**Fig. S1. Characterization of single-cell sequencing data from HSV-infected brain.**
(**A**) Representative image of head swelling of mock or HSV-1 infected mice at day 4, 6 and 8 p.i. (n=10 pr. group). (**B**) Graphical illustration of the localization of the areas in the brain stem and cerebellum subjected to GeoMx RNA analysis. An example of ROI (squares and the name of the group used in table S1) within the 2 mm tissue cores (in green circle) used for GeoMx are shown. Only ROIs with HSV1+ and CD45+ staining in the brainstem was selected for GeoMx, based on expression of HSV-1 staining on a sequential sections from the infected mice. For cerebellum ROIs selected based on CD45+ staining. Finally, we used for infected group (n=9 mice, n= 38 ROIs) and for mock group (n=3mice, 8 ROI) (**C**) An example of segmentation of ROIs s (shown in B) for GeoMx analysis of infected and control mice. The upper panels: Fluorescent labelled antibodies CD45 (red) the lower panels showing the segmentations divided into CD45+ (purple) and CD45- (gray). Lower panels: HSV-1 DAB-staining on a sequential section is shown in brown. (**D**) Scatter plot depicting the percentage of identified antisense transcripts in single-cell sequencing data across various experimental conditions. Error bars represent the standard error of the mean (SEM). Statistical significance was assessed using the Kruskal–Wallis test. (**E**) Proportion of variations in transcripts compared to reference genomic sequences. HSV−/HSV+ indicates cells without or with virus RNA, respectively. (**F**) Histogram with density curve of correlation between host gene expression and viral gene count at each time point post infection. Right panel: Expression of *Il1rn* across cell types is shown in violin plot. (**G**) Violin plots of selected cell type markers of each annotated cell type. (**H**) Absolute counts of brain-resident cell types per mouse. Error bars represent SEM across replicates. (**I**) Differences in cell type proportions of the indicated annotated cell types over time in the dataset. Data were analyzed using two-tailored one-way ANOVA.

**Fig. S2. Pathway enrichment in different cell types in the HSV-1-infected mouse brain.**
(**A-F**) Enriched GO terms for upregulated genes across different time points post-infection in the indicated cell type. (**G**) Immunohistological staining with anti-CD3 on brain stem sections from mice infected for 5 and 8 days with HSV-1.

**Fig. S3. Analysis of microglia subpopulations during HSV-1 infection.**
(**A**) Network plot illustrating the linkages between genes and biological concepts derived from enriched GO terms of upregulated DEGs at various infection time points. (**B**) Network plot illustrating the linkages between genes and biological concepts derived from enriched GO terms of downregulated DEGs at various infection time points. (**C-E**) Comparison of enriched functional terms of upregulated genes in microglial subpopulations prevalent in control, day 6, and day 8 samples, respectively. (**F**) Network plot illustrating linkages between genes and biological concepts derived from enriched GO terms in subpopulations 6, 4, and 17. (**G**) Highlight of selected genes within the enriched terms for microglia subpopulations 6, 14, and 17. (**H**) Heatmap illustrating the expression of phenotype marker genes as described [79] cross subpopulations of microglia. (**I**) Left: Enriched functions of downregulated genes in microglia from the brains of infected mice. Right: Expression of gliogenesis-related genes in microglia under different conditions and across time point or microglia subpopulations.

**Fig. S4. Analysis of monocyte subpopulations in the HSV-1-infected mouse brain.**
(**A**) UMAP of dimensional reduction of monocyte subpopulations labeled by subclustering identity and sample time points respectively. (**B**) Feature plots of marker genes of monocyte subtypes. (**C**) Dot plots showing enriched activated and suppressed terms from GSEA analysis in monocyte subpopulation 4. The network plot (right) illustrating the linkages between genes and biological concepts derived from GSEA terms in subpopulation 4. (**D**) Enriched function terms in upregulated genes in subpopulations prevalent on day 6 post infection. (**E**) Dot plots

showing enriched activated and suppressed terms from GSEA analysis in monocyte subpopulation 7. (**F**) Enriched function terms in upregulated genes in subpopulations prevalent on day 8 post infection. (**G**) Dot plots showing enriched activated and suppressed terms from GSEA analysis in subpopulation #10. (**H**) The network plot illustrating the linkages between genes and biological concepts derived from GSEA terms in subpopulation 10. (**I**) UMAP as in Fig 4E, visualizaized colored by time points to illustrate temporal progression (**J**) UMAP labeled with RNA velocity of single cells, estimated by difference between unspliced and spliced mRNAs. (**K**) PAGA map estimating directed connectivity using RNA velocity. (**L**) Ridgeline plots of pseudotime index in monocyte subpopulations.

**Fig. S5. Analysis of interactions of immune cells in the central immune system.**
(**A**) Dynamic proportions of T cell and BAM after infection. Data were analyzed using two-tailored one-way ANOVA. (**B**) Heatmap showing the prevalence of T cell and border-associated macrophage (BAM) subpopulations at the different time points. The numbers on the left indicate the subpopulation's rank, with lower numbers indicating higher abundance and higher numbers indicating lower abundance. (**C**) Bar plots visualizing the presence of different types of T cell based on projection onto the mouse T cell atlas. (**D**) Contribution of specific ligand-receptor pairs to the selected signaling pathway in predominant communication subpopulations on day 8 post infection. (**E**) Hierarchy plot visualizing signaling communications between cell groups. (**F**) Violin plots illustrating gene expression distribution of CXCL, ICOS and JAM signaling in different cell type. (**G**) Outgoing and incoming communication pattern indicates of selected cells from various time points, and the genes involved in the communication patterns.

**Fig. S6. Analysis of endothelial cells in the HSV-1-infected mouse brain.**
(**A**) Scatter plot showing the correlation between DEGs from merged single-cell transcriptome profiles and GeoMx spatial profiles on day 8 post-infection. (**B**) Network of linkages between genes and biological concepts from enriched downregulated DEGs in endothelial cells. (**C**) Plots of receptor and ligand communication pairs differentially expressed under various conditions having endothelial cells as receivers. The red and blue dot plots represent per-sample pseudobulk expression of communication pairs. (**D**) Plots of the most differentially expressed receptor-ligand communication pairs between monocytes as sender cell and endothelial cells as receivers during the course of HSV-1 infection. The same color coding is used as in panel C. (**E**) UMAP of dimensional reduction of endothelial cell subpopulations labeled by subclustering identity and sample time points respectively. (**F**) Heatmap of characteristic marker genes for endothelial cell subtypes and associated phenotypes. (**G**) Dot plot of viral transcripts in subpopulations of endothelial cells. (**H**) UMAP overlay showing *Gsdme* expression, with astrocytes labeled.

**Fig. S7. Analysis of oligodendrocyte subpopulations.**
(**A**) Network plot illustrating the linkages between genes and biological concepts derived from enriched GO terms of downregulated DEGs in oligodendrocytes. (**B**) Network plot illustrating the linkages between enriched GSEA concepts of GeoMx profiles of non-immune ($CD45^-$) cells versus control brainstem cells. (**C**) Comparison of oligodendrocyte cell type proportions across different infection time points. (**D**) Dot plot showing the expression patterns of selected genes in subpopulations. (**E**) Comparison of enriched functional GO terms for upregulated genes in subpopulations across different conditions. (**F**) Top: UMAP representation of the inferred trajectory, labeled by subpopulation identity. Bottom: UMAP of the inferred trajectory,

colored by pseudotime progression. (**G**) Ridgeline plots of pseudotime index in each oligodendrocyte subpopulation. (**H**) UMAP labeled with trajectory paths inferred by slingshot and RNA velocity of single cells. (**I**) Heatmap showing modules of co-regulated genes, with selected subpopulations labeled. (**J**) UMAP showing expression of gene modules 28 and 9 from fig.S7I. Gene modules 28 and 9 are uniquely expressed in subpopulation 4 and 9, respectively. (**K**) Comparison of enriched functions of genes expressed in modules 28 and 9. (**L**) Expression of selected gene modules displayed on UMAP. (**M**) Heatmap showing the regulon activity scores of oligodendrocyte subpopulation-specific transcription factors. (**N**) Incoming communication patterns of cells from the top communicating subpopulations.

**Fig. S8. Analysis of microglia and monocyte subpopulation representation in published datasets.**
(**A**) UMAP of the JEV dataset, labeled with various symptoms and transferred cell type identities from the HSV-1 dataset analyzed in the present work. (**B**) Comparison of cell proportions in selected subpopulations across different conditions in JEV dataset. (**C**) Comparison of cell proportions in selected subpopulations across different conditions in TBI dataset. (**D-G**) TWEAK signaling pathway genes expression pattern in oligodendrocyte and microglia from EAE, TBI, GBM and AD datasets. (**H**) *Tnfsf12* gene expression pattern in microglia subpopulations from the listed datasets.
